# Meta‐GWAS of Pig Semen Quality Traits Reveals Conserved Genes Regulating Mammalian Fertility

**DOI:** 10.1002/advs.202515203

**Published:** 2026-01-08

**Authors:** Qing Lin, Xiaodian Cai, Zhanming Zhong, Tingting Li, Xinyou Chen, Wondossen Ayalew, Zhiting Xu, Chen Wei, Xiaoke Zhang, Hong Cheng, Zhenyang Zhang, Xuehua Li, Yongjie Tang, Siqian Chen, Jun Zhou, Jinglei Si, Xibo Wu, Chao Ning, Qishan Wang, Yuchun Pan, Yahui Gao, Jiaqi Li, Ying Yu, Zhe Zhang, Yunxiang Zhao, Lingzhao Fang, Zhe Zhang

**Affiliations:** ^1^ State Key Laboratory of Swine and Poultry Breeding Industry Guangdong Laboratory for Lingnan Modern Agriculture, Guangdong Provincial Key Lab of Agro‐Animal Genomics and Molecular Breeding College of Animal Science South China Agricultural University Guangzhou China; ^2^ Center for Quantitative Genetics and Genomics Aarhus University Aarhus Danmark; ^3^ Department of Animal Science College of Animal Science Zhejiang University Hangzhou China; ^4^ Guangxi Key Laboratory of Animal Breeding Disease Control and Prevention College of Animal Science and Technology Guangxi University Nanning China; ^5^ National Engineering Laboratory for Animal Breeding State Key Laboratory of Animal Biotech Breeding Breeding and Reproduction of the Ministry of Agriculture and Rural Affairs College of Animal Science and Technology China Agricultural University Beijing China; ^6^ Guangxi State Farms Yongxin Animal Husbandry Group Co., Ltd. Nanning China; ^7^ Shandong Provincial Key Laboratory For Livestock Germplasm Innovation & Utilization College of Animal Science Shandong Agricultural University Tai'an China

**Keywords:** integrative genomics, meta‐GWAS, pig, PigGTEx, semen quality

## Abstract

Semen quality serves as a vital indicator of male fertility, yet its underlying genetic and regulatory mechanisms remain poorly understood. Here, 1.15 million records of six semen quality traits from 14 210 boars in four distinct breeds were collected. These traits have low to moderate heritability (0.12–0.26), and are genetically correlated with growth traits like average daily gain. Genome‐wide association study (GWAS) and multi‐breed meta‐analysis detected 234 loci associated with semen quality. Systematic integration of the Pig Genotype‐Tissue Expression resource with these GWAS loci allowed the prioritization of 93 causal variants targeting 134 genes. For instance, the expression quantitative trait loci (eQTL) of *NAXE* in multiple tissues were colocalized with a GWAS loci of the number of sperms, while eQTL of *LEFTY2* in the testis was exclusively colocalized with in a GWAS loci of semen volume. Through examining GWAS of semen quality traits in cattle and human complex traits, the ortholog genes (e.g., *AURKAIP1* and *ADRA2A*) significant in pigs also regulated bovine semen quality, and were significantly enriched for heritability of human birth weight and height. This study provides novel insights into semen quality traits in mammals, which will provide candidate genes for pig selective breeding and potential targets for human male infertility research.

## Graphical abstract

This study incorporated 14 210 individuals to perform a GWAS meta‐analysis of six semen quality traits. The GWAS meta‐analysis identifies 234 GWAS loci associated with semen quality traits. The incorporation with a functional genomics resource explains potential genetic regulation of ∼40% GWAS signals underlying semen quality traits. Cross‐species analysis discovers the potential links between human, cattle, and pigs in semen quality traits.

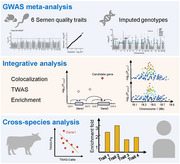

**Abbreviations**
EBVs: estimated breeding values
eQTL: expression quantitative trait loci
FAANG: Functional Annotation of Animal Genomes
GWAS: Genome‐wide association study
LD: linkage disequilibrium
LMA_100: Loin muscle area (100 kg)
molQTL: molecular quantitative trait loci
NMSP: number of motile sperms
NSP: number of sperms
PigGTEx: Pig Genotype‐Tissue Expression
PP4: Posterior probability
SEMVOL: semen volume
SPABR: sperm abnormality
SPCON: sperm concentration
SPMOT: sperm motility
TWAS: transcriptome‐wide association studies
WGS: whole‐genome sequence

## 1 Introduction

Beyond their important roles in the global agri‐food system, pigs serve as one of the crucial biomedical models for understanding human biology and disease [1]. In the pig and cattle breeding industry, male fertility is a trait of economic importance (e.g., reproductive efficiency and breeding progress), while in humans, male infertility represents a significant health and social problem. Yet, the genetic and regulatory mechanisms underlying male fertility remain poorly understood [2]. Severe oligozoospermia and azoospermia conditions are characterized by low or absent sperm count, that often caused by complex genetic factors [3]. Semen quality is thus one of the most important indicators of male fertility. In farm animals like pigs and cattle, males play an indispensable role in the breeding and production industry, especially with the widespread adoption of artificial insemination techniques [4]. For example, one boar could mate with a limited number of sows (e.g., 20–30 per year) in natural mating, whereas it can provide enough semen for about 150 doses of semen through artificial insemination. Understanding the genetic regulation of semen quality traits is beneficial to reduce feeding costs and improve the semen quality in of boars. Therefore, deciphering the genetic architecture of semen quality traits is vital in addressing male infertility in humans and enhancing reproductive efficiency in farm animals.

Genome‐wide association studies (GWAS) have discovered many loci associated with semen quality traits across various species, including humans (sample size ranging from 269 to 1590) [5, 6, 7], cattle (1390 and 1508) [8, 9], pig (129, 583, 1733 and 3596) [10, 11, 12, 13], and sheep (429) [14], which provided a general insight to understand the genetic architecture of semen quality traits. However, these studies were limited in sample size and signal populations. In addition, the detected variants were located in the non‐coding genomic region [15], making it challenging to interpret the underlying molecular mechanisms due to the linkage disequilibrium (LD) [16] and intricate regulatory processes [15, 17]. To address these limitations, integrative multi‐omics approaches have emerged, which provided an chance to systemically explore the molecular mechanisms underlying complex traits. For instance, integrating GWAS with functional genomic resources, such as ENCODE [18], Roadmap Epigenomics Program [19], and Genotype‐Tissue Expression (GTEx) project [20], provides novel insights into the regulatory mechanisms underlying complex traits in humans. Similarly, emerging international efforts, such as the Functional Annotation of Animal Genomes (FAANG) project [21] and FarmGTEx project [22, 23, 24, 25], provide valuable resources to explore the genetic mechanisms underlying complex traits in farm animals. For example, the integration of GWAS and PigGTEx resource could interpret the genetic regulation of complex traits in pigs [26]. These efforts enable a better understanding of the genetic regulation underlying semen quality traits in pigs.

**FIGURE 1:**
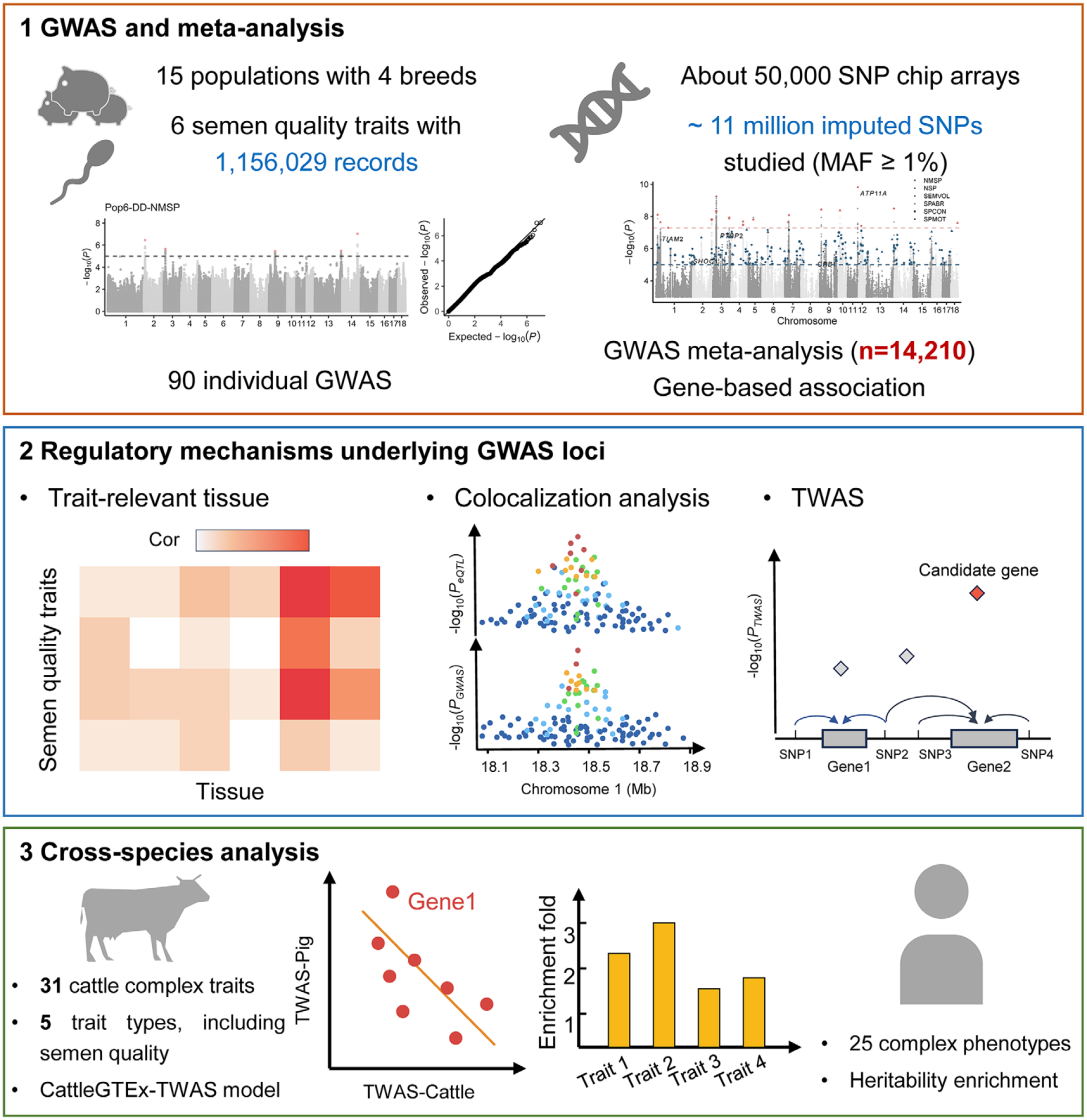
The workflow for interpreting the genetic regulatory mechanism of semen quality traits in pigs. The first layer presents information on GWAS populations, including the sample size, breeds, genotypes, phenotypes, individual GWAS and GWAS meta‐analysis. The second layer indicates the trait‐tissue enrichment, colocalization analysis and transcriptome‐wide association study (TWAS). The third layer represents the cross‐species analysis for semen quality traits between human, cattle and pig.

In this study, to explore the genetic and molecular architecture underlying semen quality in pigs, we collected genotypes and six semen quality traits (including 1 156 029 records) in 14 210 pigs from 15 different populations in four distinct breeds, including Duroc, Landrace, Yorkshire, and Pietrain (Figure 1). To improve the resolution of genetic variants, we imputed genotypes to the whole‐genome sequence (WGS) level and conducted GWAS within each of four breeds, followed by multiple‐breed meta‐analysis and gene‐based association analysis. Furthermore, we systematically integrated these GWAS findings with chromatin states of 14 porcine tissues in FAANG and molecular quantitative trait loci (molQTL) of 34 pig tissues in PigGTEx to prioritize the potential causal variants, genes, and tissues. By examining the GWAS of semen quality traits in cattle, we explored the conservation of the genetic and molecular basis of semen quality between mammals. Ultimately, we explored whether orthologs of these detected genes are associated with a range of human complex traits and diseases.

## 2 Results

### 2.1 Genetic Parameters of Semen Quality Traits

**FIGURE 2:**
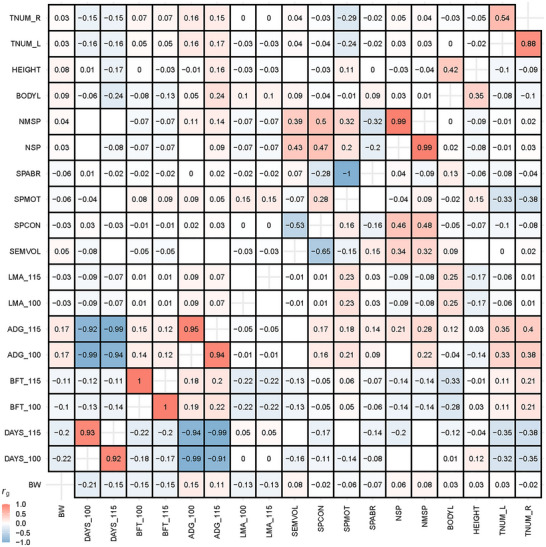
The genetic and phenotypic correlation between semen quality traits and growth, and body type traits in pigs. The upper triangle indicated the phenotypic correlation. The lower triangle indicated the genetic correlation. The white boxes located in non‐diagonal elements indicates the missing values.

To explore the genetic basis of semen quality, we first estimated the genetic parameters (including narrow‐sense heritability, repeatability, and genetic correlation) for six semen quality traits, including semen volume (SEMVOL), sperm abnormality (SPABR), sperm concentration (SPCON), sperm motility (SPMOT), number of sperms (NSP), and number of motile sperms (NMSP), in each of 15 populations (Table S1 and Figure S1). The heritability of semen quality traits was varied across breeds and populations, ranging from 0.12 to 0.26 (*h^2^*_NSP_ = 0.13 ± 0.04, *h^2^*_NMSP_ = 0.12 ± 0.04, *h^2^*_SEMVOL_ = 0.23 ± 0.08, *h^2^*_SPABR_ = 0.26 ± 0.17, *h^2^*_SPCON_ = 0.21 ± 0.06, and *h^2^*_SPMOT_ = 0.21 ± 0.13) (Figure S2A and Table S2). The estimated repeatability of semen traits ranged from 0.29 to 0.44 (Figure S2A). In addition, genetic correlation was varied among semen quality traits, ranging from ‐0.84 to 0.99 (Figure S2B). For example, NSP was highly correlated with NMSP (*r*_g[NSP, NMSP]_ = 0.98 ± 0.01), while SPABR was lowly correlated with SEMVOL (*r*_g[SEMVOL, SPABR]_ = 0.11 ± 0.12). We further explored the genetic correlation between semen quality traits and other types of traits (ranging from ‐0.38 to 0.28), including productive, reproductive, and body type traits (Figure 2). The results showed that the average daily gain had a positive genetic correlation with semen quality traits, particularly SPMOT (*r*_g[ADG_100, SPMOT]_ = 0.21 ± 0.03) and NMSP (*r*_g[ADG_100, NMSP]_ = 0.22 ± 0.03) (Figure 2). In contrast, the backfat thickness at 100 kg (BFT_100) showed a negative genetic correlation with SEMVOL (*r*_g[BFT_100, SEMVOL]_ = ‐0.12 ± 0.04) (Figure 2).

### 2.2 Single‐Breed GWAS Analysis

We obtained an average imputation accuracy of 97.50% across populations (Figure S3). To investigate the genetic architecture of semen quality, we conducted the individual GWAS for each semen quality trait within each of the 15 populations using the repeatability model implemented in GMAT (v1.02) [27]. The analysis yielded 90 individual GWAS summary statistics for six semen quality traits with an average genomic inflator (lambda) of 0.97 across populations and traits (ranging from 0.82 to 1.08, Figure S4 and Table S3). We conducted the conditional analysis to detect independent signals based on the individual GWAS summary statistics using GCTA‐COJO [28]. We discovered a total of 344 suggestive significant loci (*p* < 1.00 × 10^−5^), corresponding to 345 independent variants across populations and semen quality traits. The number of independent variants positively correlated with sample size across six semen quality traits (Figure S5A). To further quantify the semen quality‐associated regions, we defined the QTL regions based on the LD (*r* ^2^ ≥ 0.6 with lead SNP) derived from corresponding populations, resulting in 345 QTL for all the semen quality traits (Table S4). Most of the QTL were novel discoveries compared to those in PigQTLdb [29] (Figure S5B). Functional annotation of 277 candidate genes within these QTL revealed enrichment in pathways related to energy metabolism and immune function (Figure S5C and Table S5), linking to spermatogenesis in male fertility [30, 31].

### 2.3 Multi‐Breed Meta‐GWAS Reveals Novel Semen Quality‐Associated Loci

**FIGURE 3:**
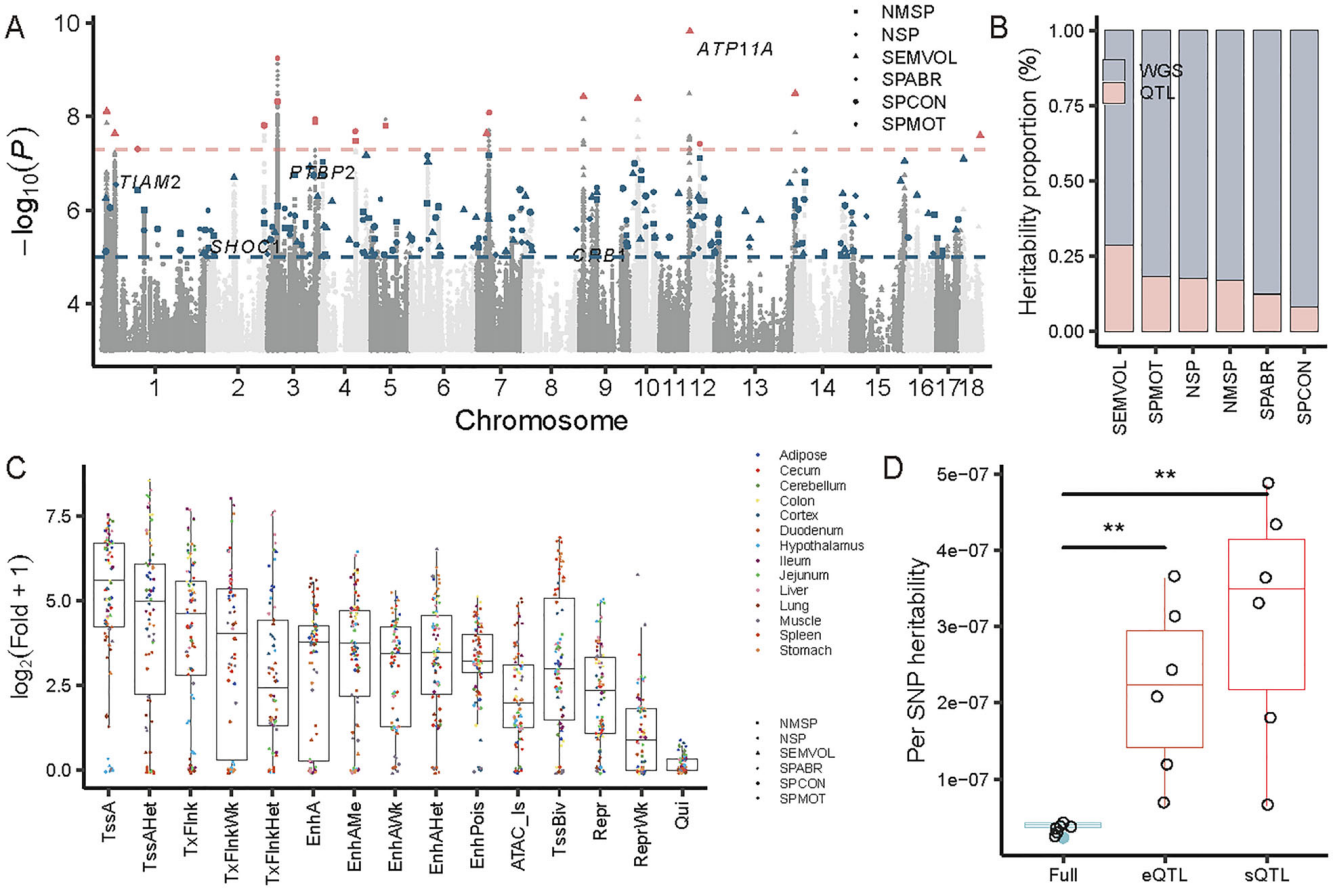
GWAS meta‐analysis discoveries the loci associated with semen quality and functional annotation for semen quality‐associated variants. (A) The Manhattan plot of GWAS meta‐analysis across six semen quality traits. The red and blue dashed lines represent the genome‐wide (*p* < 5.00 × 10^−8^) and suggestive (*p* < 1.00 × 10^−5^) significant threshold, respectively. The red and blue points represent genome‐wide and suggestive significant independent variants deriving from conditional analysis. The shape of points represents different semen quality traits. (B) The distribution of heritability proportion explained by QTL across six semen quality traits. ‘WGS’ (red) and ‘QTL’ (blue) indicate the variants across all chromosomes and only located in the QTL, respectively. The bars are sorted by the proportion of heritability explained by QTL. (C) The heritability enrichment explained by corresponding regulatory elements across 14 tissues. TssA, Strongly active promoters/transcripts. TssAHet, Flanking active TSS without ATAC. TxFlnk, Transcribed at gene. TxFlnkWk, Weak transcribed at gene. TxFlnkHet, Transcribed region without ATAC. EnhA, Strong active enhancer. EnhAMe, Medium enhancer no ATAC (hetero). EnhAWk, Weak active enhanver. EnhAHet, Active enhancer no ATAC (hetero). EnhPois, Poised enhancer. ATAC_Is, ATAC island. TssBiv. Bivalent/poised TSS. Repr, Repressed polycomb. ReprWk, Weak repressed polycomb. Qui, Quiescent. (D) The per SNP heritability explained by different variants. The significant test was conducted by the ggsignif R package. The “^**^” indicated the *p* value < 0.01.

To ensure the reliability and robustness of meta‐GWAS, we performed quality control on individual GWAS summary statistics using EasyQC v23.8 [32] before the meta‐analysis. The results indicated a negative correlation between sample size and the median standard error (Figure S6A,B). We then conducted the meta‐GWAS analysis by combining the individual GWAS of each semen quality trait across populations and breeds using an inverse‐variance weighted fixed‐effects model by METAL (released on May 5, 2020) [33]. In total, the meta‐analysis identified 4807 variants significantly (*p* < 1.00 × 10^−5^) associated with six semen quality traits. Conditional analysis identified 234 suggestively significant loci (including 19 genome‐wide significant loci, *p* < 5.00 × 10^−8^), corresponding to 237 independent variants (20 genome‐wide significant) (Figure 3A, Table S6). Comparing with individual GWAS, 28.4% GWAS signals (98 out of 345 signals) could be replicated in the GWAS meta‐analysis. Notably, the closest genes, such as *TIAM2* [34], *PTBP2* [35], and *ATP11A* [36], were previously reported to be associated with spermatogenesis and early embryonic development.

To explore the candidate genes associated with semen quality traits, we utilized the meta‐GWAS summary statistics to perform the gene‐based association analysis using MAGMA [37]. This analysis yielded 82 suggestive (Bonferroni correction for the number of tested genes, *p* < 4.37 × 10^−5^) significant gene‐trait pairs (Figure S7 and Table S7). Among the significant genes, the *CYP26B1* gene was linked to germ cell differentiation by degrading retinoic acid [38, 39], and the *NFIX* gene was involved in meiotic progression during the first wave of spermatogenesis [40]. These findings provide valuable insights into the potential candidate genes in post‐GWAS analysis.

To investigate the potential shared loci between semen traits and other types of traits in pigs, we performed the GWAS, conditional analysis, and gene‐based association analysis for 13 growth and body type traits (Methods). The lead SNP (7_30238221_T_C) of Loin muscle area (100 kg) (LMA_100) was found to be colocalized (PP4 = 0.51) with semen quality traits (including SEMVOL, NSP, and NMSP) with candidate gene *SMIM29* on chromosome 7 (Figure S8A–C). Additionally, several genes were significantly associated with both semen quality traits (including NSP and NMSP) and LMA_100 (Figure S9A,B), indicating a shared regulatory mechanism between semen quality and growth traits. To validate the discovery of semen quality‐associated loci, we compared the results from meta‐GWAS with those from the PigQTLdb. The meta‐GWAS showed a greater overlap of QTL with the PigQTLdb than individual GWAS (Figure S10A; Figure S5B). Additionally, 19.4% GWAS signals (seven out of 36 signals) derived from Cheng's study [13] could be replicated (Fisher test odds ratio = 0.18, *p* = 4.49 × 10^−4^, Figure S10B). Using the population with the largest sample size (3607 individuals), we partitioned heritability using a two‐component model (Methods), and found that the identified QTL explained an average of 17.11% of the heritability across six semen quality traits (Figure 3B). Functional annotation results showed that the candidate genes were enriched in the chromatin structure, DNA binding, and immune response terms (Figure S10C and Table S8).

### 2.4 Semen Quality‐Associated Variants Enriched in Regulatory Elements and Variants

As expected, a large proportion of variants associated with semen quality were located in the non‐coding regions, including intron (44.90%) and intergenic (28.20%) (Figure S10D), which suggests a complex regulatory mechanism underlying semen quality traits. To further explore the functional implications of these variants, we incorporated the chromatin state information across 14 tissues [41] to estimate the per SNP heritability (Methods). The results revealed that active promoter and enhancer regions accounted for a higher per SNP heritability compared to the rest of chromatin states (Figure 3C). Furthermore, we observed that gene expression and splicing QTL (e/sQTL) data [23] from the PigGTEx explained a significantly higher per SNP heritability than other variants (Figure 3D), indicating that the regulatory variants played a vital role in the genetic regulation of semen quality traits. Additionally, the heritability estimates derived from e/sQTL varied between tissues (Figure S11A,B), indicating a complex regulatory mechanism that operates in a tissue‐specific manner. Notably, the early developmental tissues, such as morula and embryo, explained the higher SNP heritability than other tissues across semen quality traits (Figure S11A,B), suggesting a strong relationship between the early developmental tissue and sperm quality.

### 2.5 Colocalization Between Multi‐Tissue eQTL and GWAS Loci

**FIGURE 4:**
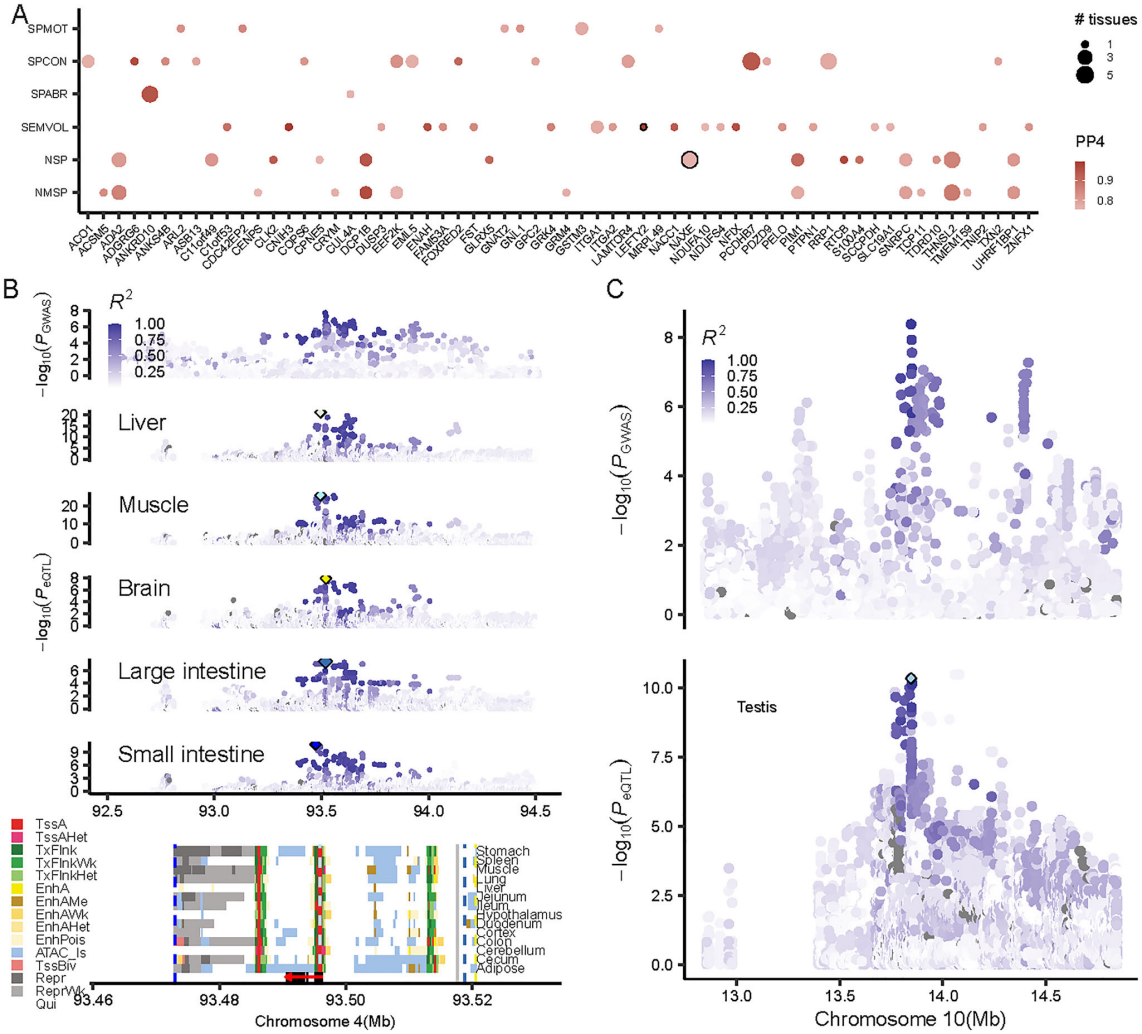
Colocalization analysis provided an insight to understand the molecular regulatory mechanism of semen quality traits. (A) The summary of colocalization analysis across six semen quality traits. PP4, posterior probability 4. (B) The locus zoom of GWAS loci with lead variant rs322657255. The top panel represents the GWAS loci. The second to fifth panels represent the eQTL mapping of *NAXE* gene with corresponding tissues labeled in the panels. The diamond points indicated the top eQTL. The bottom panel represents the chromatin states across 14 tissues. The black boxes at the bottom indicate the gene body. The red arrow indicates the transcript direction of the gene. (C) The locus zoom of GWAS loci with lead variant rs81420828. The top panel represents the GWAS loci. The bottom panels represent the eQTL mapping of *LEFTY2* gene with corresponding tissues.

To explore whether eQTL and GWAS loci share the same genomic variants, we integrated the PigGTEx resource with meta‐GWAS by implementing colocalization. In total, 39.29% (93 out of 237 significant loci) were explained by gene‐based association, colocalization, and transcriptome‐wide association study (TWAS) (Figure S12). Colocalization analysis yielded 121 significant gene‐tissue‐trait pairs (PP4> 0.75, Figure 4A and Table S9), with some pairs playing a crucial role in semen quality through various regulatory mechanisms. For example, the eQTL of the *NAXE* (*AIBP*) gene was colocalized with NSP across multiple tissues, including the brain, intestine, muscle, and liver (Figure 4B; Figure S13A). The top eQTL in the colocalized tissues had a high linkage disequilibrium (*r* ^2^ = 0.87). Specifically, the top eQTL in the liver (*rs326817713*, missense variant of *NAXE*, *r* ^2^ = 0.82) and muscle (rs339011837, upstream gene variant of *NAXE*, *r* ^2^ = 0.82) are located in the TssA (Strongly active promoters/transcripts) region of *NAXE*, indicating its important role in regulating NSP via changing gene expression. In addition, the other tissues, including the brain (rs319126653, intron variant of *IQGAP3*, *r* ^2^ = 0.99), small intestine (rs341354982, intergenic variant, *r* ^2^ = 0.82), and large intestine (rs336133524, intron variant of *IQGAP3*, *r* ^2^ = 0.90), also colocalized with NSP in the *NAXE* gene, indicating the complexity in the regulatory mechanism underlying semen quality. Recent studies have linked the *NAXE* gene to protein phosphorylation and cholesterol efflux during sperm capacitation [42]. However, the ubiquitous expression of the *NAXE* gene (Figure S13B) made it difficult to pinpoint its regulatory role in terminal complex phenotypes [43]. These results indicated that the *NAXE* gene regulates the NSP through various tissues rather than the terminal tissue (such as the testis and sperm). To further explore the regulatory mechanism of these loci, we conducted a conditional analysis and identified a secondary signal with lead SNP (rs343458850, intron variant, *p* = 5.50 × 10^−5^, Figure S12C).

The eQTL of the *LEFTY2* (TGF‐β) gene in the testis only was colocalized with SEMVOL, although it had eQTL in blood and testis (Figure 4C). The *LEFTY2* gene has been identified as a potential regulator that may reinforce the male fate and prevent meiosis in embryonic germ cells [44]. We found that the *LEFTY2* gene was high‐expressed in the testis across 34 tissues (Figure S14A). In addition, the top eQTL of the *LEFTY2* gene in testis (*rs81420828*, downstream gene variant of the *LEFTY2* gene, Figure S14B) colocalized with the lead SNP of SEMVOL, located in its TssA (Strongly active promoters/transcripts) region. These results indicated that colocalization analysis could identify genetic variants with putative genes associated with semen quality traits mediated by gene expression in multiple tissues.

### 2.6 TWAS Detected Additional Semen Quality Traits‐Associated Genes

**FIGURE 5:**
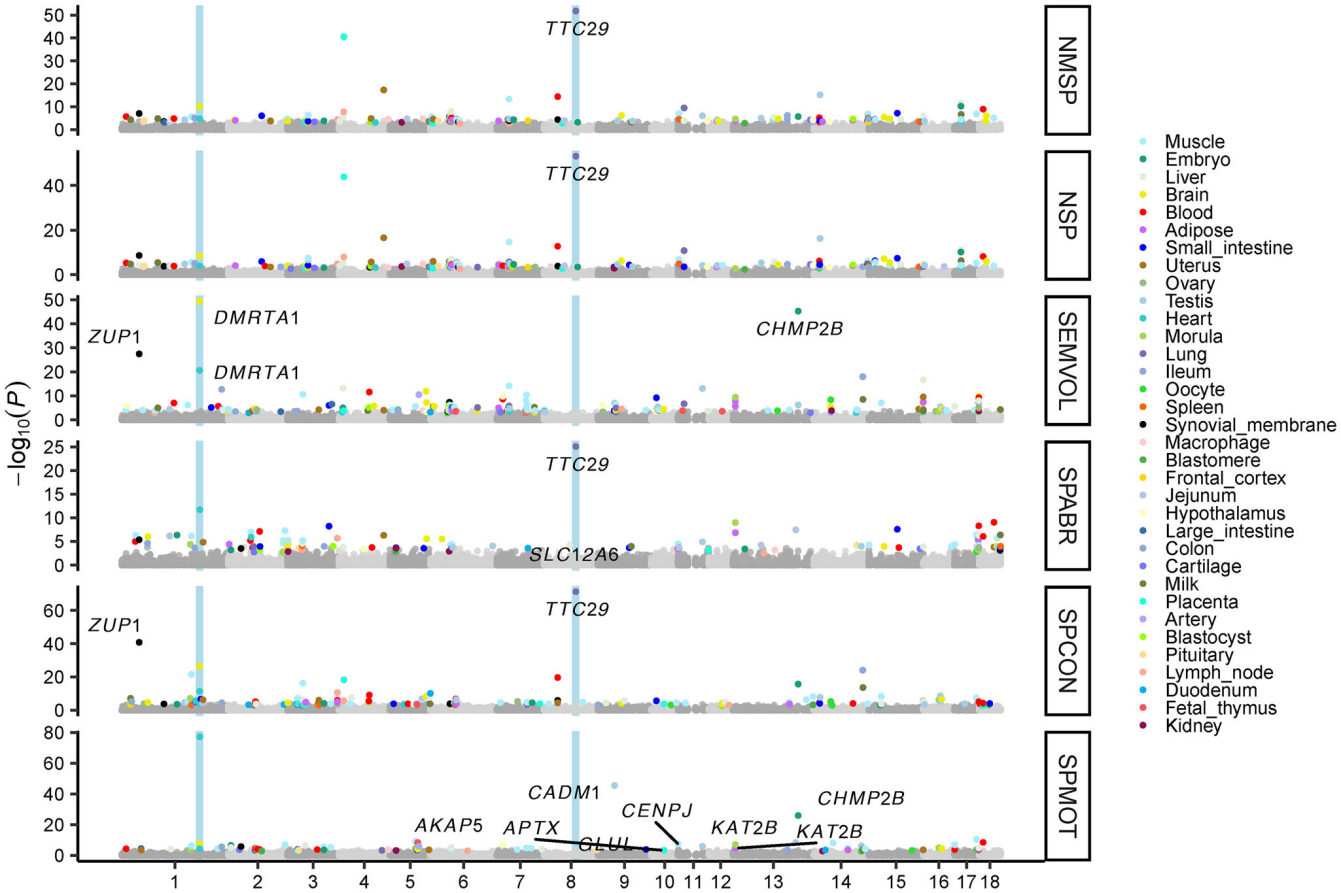
TWAS detected additional discovery of candidate genes associated with semen quality traits.

To further investigate the potential causal genes associated with semen quality traits, we conducted transcriptome‐wide association studies (TWAS). A total of 909 significant gene‐tissue‐trait pairs (Bonferroni correction for tested genes for each tissue) were detected, representing six semen quality traits and 34 tissues (Figure 5, Table S10). For example, although the *TTC29* gene has no eQTL in the lung, it was the most significant gene of semen quality traits (including SPCON, SPABR, NMSP, and NSP) in this tissue (Figure 5). Furthermore, the gene expression level of the *TTC29* gene in the testis was the highest among all 34 tissues (Figure S15A), and it showed the highest expression in spermatocytes across all 261 cell types in the testis (Figure S15B). Previous studies have shown that the mutations in the *TTC29* gene resulted in multiple morphological abnormalities of the flagella in humans [45, 46, 47], leading to male infertility. However, the TWAS results in the testis were not significant for the *TTC29* gene, which might be due to the small sample size in eQTL mapping.

In addition, the *DMRTA1* gene was significantly associated with all the semen quality traits except SPABR in the brain and heart (Figure 5). The *DMRTA1* gene was highly expressed in the pituitary testis, spermatocyte (one of the cell types in the testis), and lactotrope (one of the cell types in the pituitary, part of the brain) (Figure S16A,B). Furthermore, previous studies showed that the *DMRTA1* gene was related to sex differentiation [48] and gonadal development [49], supporting its role in sexual differentiation and germ cell development. In conclusion, beyond colocalization analysis, our TWAS results provide additional insights into the regulatory mechanisms underpinning semen quality traits.

### 2.7 Comparative Analysis Revealed Conserved Genes of Semen Quality Traits in Mammals

**FIGURE 6:**
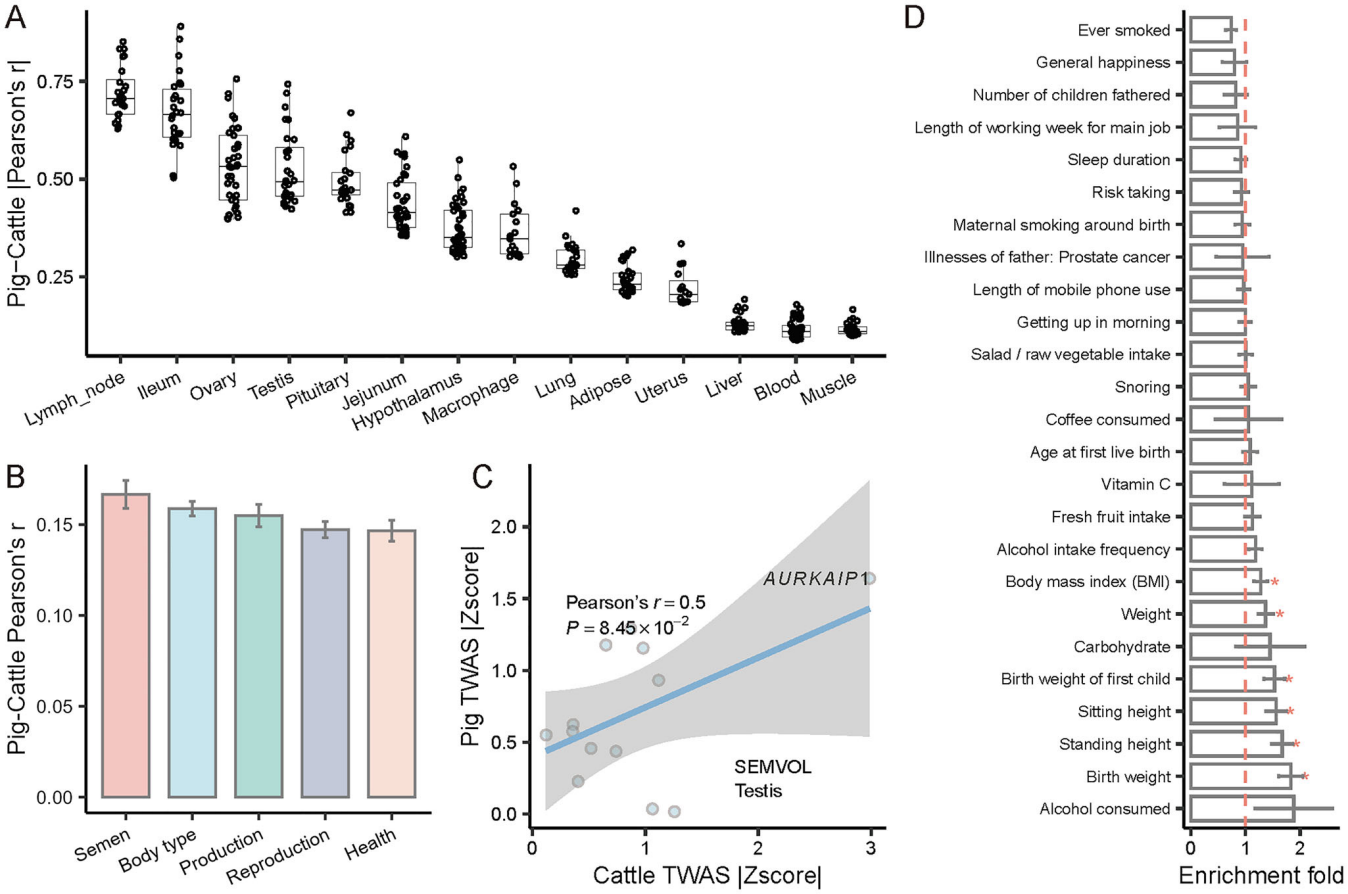
Cross‐species comparative analysis indicating species conservation in pig, cattle and human. (A) The Pearson's correlation of absolute z‐score derived from TWAS between cattle and pig across 14 tissues. Each point represents a trait pair. (B) The average of Pearson's correlation of absolute z‐score derived from TWAS between cattle and pig for each trait group. The error bar indicated the corresponding standard error. (C) The average of Pearson's correlation of absolute z‐score derived from TWAS between cattle and pig in SEMVOL/Testis. The blue line is linear by the geom_smooth function. (D) The heritability enrichment across 25 complex traits/diseases in humans. The red dashed line indicates the enrichment fold = 1. The red stars indicated significant enrichment (fold>1 and *p* value < 0.05). The error bar indicated the corresponding standard error.

By incorporating the cattle TWAS obtained from the CattleGTEx resource (including 48 traits with 15 tissues), we calculated the Pearson's correlation of the absolute z‐score of complex traits for the one‐to‐one orthologous genes in the same tissue between pigs and cattle (Table S11). Among all the traits, the Pearson's correlations of semen quality traits between pigs and cattle were highest (Figure 6A,B), indicating the conservation of semen quality regulation between pigs and cattle. Several tissue pairs exhibited a high Pearson's correlation between pigs and cattle (Figure S17A), such as SEMVOL in the testis (Figure 6C). The *AURKAIP1* gene was associated with semen volume in both pig and cattle (Zscore_pig_ = 1.47, Zscore_cattle_ = 1.79), which was involved in male meiotic prophase [50]. Additionally, SPMOT was significantly correlated (Pearson's *r* = 0.51, *p* = 2.94 × 10^−2^, Figure S17B,C) in the jejunum between cattle and pigs. The *ADRA2A* gene was associated with SPMOT in cattle and pigs (Zscore_pig_ = 1.64, Zscore_cattle_ = 2.99), which was already associated with sperm‐specific functions such as sperm capacitation, acrosome reaction, and motility [51]. To further investigate the functional impacts of these semen quality trait‐associated genomic regions in humans, we conducted the heritability enrichment analysis of their orthologous regions in 25 human complex traits/diseases using LDSC [52]. These orthologous regions were significantly enriched in the reproductive trait (such as birth weight and birth weight of first child) and body type traits (sitting/standing height and BMI) in humans (Figure 6D). Furthermore, these regions were enriched in the cancer of other male genital organs and type 2 diabetes, while it was not statistically significant. In contrast, it was not enriched in other phenotypes, such as ever smoked and general happiness. These results indicated that pigs might be a potential model for studying human male fertility and relevant complex traits and diseases.

## 3 Discussion

In this study, we comprehensively explored the genetic parameters of semen quality traits in pigs and investigated their genetic correlations with productive, reproductive, and body size traits. Our individual GWAS and meta‐GWAS provided essential insights into understanding the genetic architecture of semen quality traits in pigs. Notably, regulatory elements/variants explained a significant portion of the heritability associated with semen quality traits. By integrating the FAANG and PigGTEx resources, we identified candidate causal genes associated with semen quality traits, such as the *NAXE* gene colocalized with various tissues and the *LEFTY2* gene specifically colocalized with the testis. Our cross‐species analysis revealed that semen quality traits exhibit higher conservation between pigs and cattle than other complex traits. Furthermore, semen quality trait‐associated genomic regions were enriched for human reproductive and morphological traits, such as birth weight, birth weight of the first child, and sitting/standing height. These findings suggest a conserved genetic basis for reproductive traits across species.

The heritability of semen quality traits was low (ranging from 0.12 to 0.26), consistent with previous studies [53, 54]. The low heritability of semen quality traits indicated that semen quality was partially influenced by environmental factors, such as feeding conditions, temperature [55], micro‐environment, and nutrition [56]. In addition, the genetic background (e.g., breed) was also a key factor influencing the semen quality. Moreover, we investigated the genetic and phenotypic correlation between semen quality traits and other types of traits, indicating the potential links between semen quality traits and growth traits in boar. Despite the importance of semen quality traits, they remain underrepresented in genomic databases. For example, the Pig QTLdb (release 52) contains a limited number of QTL associated with semen quality, highlighting the need for further exploration. To increase the sample size of GWAS analysis, we incorporated 15 populations, including 14 210 individuals, to perform GWAS meta‐analysis, which identified 234 GWAS signals. Meanwhile, a small proportion of GWAS signals could be found in previous study, while this study yielded a large proportion of new GWAS signals, filling a critical gap in our understanding of these traits.

By incorporating data from multiple breeds, we enhanced the resolution of our analyses, reducing linkage disequilibrium and fine‐mapping potential causal genes. Previous studies have primarily focused on identifying candidate genes associated with semen quality traits [10, 11, 12], while they have often overlooked the underlying molecular regulatory mechanisms. Our approach of integrating functional genomics data enabled the fine mapping of the potential causal genes and investigated the regulatory mechanism of semen quality traits. This integrative strategy is crucial for unraveling the complex genetic architecture of semen quality traits. Furthermore, the cross‐species comparative analysis showed the potential shared genetic regulation of semen quality traits between cattle and pigs. The heritability enrichment on human complex traits also showed the potential genetic conservation between growth traits and semen quality traits. However, the potential links between semen quality traits in humans and pigs have been uninvestigated because of the absence of semen quality traits data in humans.

Although the regulatory variants of gene expression could explain about 40% of GWAS loci, a significant proportion remains unaccounted for, suggesting the involvement of complex regulatory processes beyond eQTL. Similarly, a previous study confirmed that the eQTL could only explain a low to median proportion of GWAS loci [57], indicating the complicated regulatory processes and the importance of additional omics information, such as chromatin accessibility (caQTL), epigenetic modifications (epiQTL) to elucidate these mechanisms. Furthermore, considering functional genomics in the context of specific cell types and sex will provide deeper insights into trait regulation. Semen quality traits are also limited by population size, which constrains the identification of trait‐associated variants. Addressing this limitation through large‐scale, multi‐ancestry studies is critical.

## 4 Conclusions

In conclusion, our GWAS meta‐analysis, combined with functional genomics, advances the understanding of the genetic architecture and regulatory mechanisms underlying semen quality traits, providing a foundation for future research and applications in livestock breeding.

## 5 Materials and Methods

### 5.1 Populations and Phenotypes

In total, we collected genotypes (most were based on SNP arrays) and 1 156 029 records of semen quality in 14 210 genotyped pigs from 15 different populations in four distinct breeds, including Duroc, Landrace, Yorkshire, and Pietrain. Individuals from the same farm and of the same breed were grouped into one population. In total, 11 populations with primary phenotype and genotype data (including 11 528 genotyped individuals) and 4 populations with GWAS summary statistics [13] were collected. For the phenotypes, four semen quality traits were measured using the CASA system that used high‐resolution phase‐contrast microscopy combined with image processing algorithms to distinguish sperm morphology. Finally, we defined six semen quality traits, including (1) semen volume (SEMVOL, ml), (2) sperm abnormality (SPABR, ranging from 0 to 1), (3) sperm concentration (SPCON, × 10^8^/ml spermatozoa per ml), (4) sperm motility (SPMOT, ranging from 0 to 1), (5) the number of sperms (NSP, × 10^8^ spermatozoa), calculated as SEMVOL × SPCON, and (6) the number of motile sperms (NMSP, 10^8^ spermatozoa), derived from NSP × SPMOT. We removed the records with (1) SEMVOL more than 500 mL; (2) SPCON higher than 20 × 10^8^/ml, and (3) the interval between two semen collections more than 30 d. Finally, we retained 1 156 029 records from 14 210 boars for subsequent analyses. The summary of phenotypic data for each semen trait in each population is in Table S1.

### 5.2 Genotypes

All the following genomic analyses were based on *Sus scrafa* 11.1 version 110. The pigs were genotyped by 50K GeneSeek GGP‐Porcine Beadchip (Neogen Corporation, Lansing, MI, USA) and “Zhongxin­I” Porcine Breeding Chip (Beijing Compass Agritechnology Co., Ltd., Beijing, China), which included 50 698 and 57 467 SNPs, respectively. For the low‐density genotypes (∼50k), we filtered out the SNPs with MAF < 0.01 and individuals with allele call rate < 0.9 in each population. Afterwards, we imputed all the genotypes to the whole‐genome sequence level using PGRP v1, which included 1602 pigs and 42 523 218 SNPs, by Beagle v5.1 [58]. After genotype imputation, we removed SNPs with MAF < 0.01 and DR^2^< 0.8. We employed two strategies to evaluate the accuracy of genotype imputation: (1) concordance rate (CR), indicating the consistency between imputed and observed genotypes, and (2) genotype correlation (*R^2^*), quantifying the squared correlation between the observed and imputed minor allele doses in target panels. We employed 20‐fold cross‐validation on the target panels among autosomes. During each set of cross‐validation, we randomly removed 5% SNPs from the target panels as a validation set and then employed Beagle v5.1 to impute them using PGRP v1.

For the concordance rate between imputed and observed genotypes, the formula was as follows:

$$CR=\frac{Genotype_{imp}}{Genotype_{obs}}$$

where *Genotype_imp_* indicates the number of imputed genotypes consistent with the observed genotypes, *Genotype_obs_* represents the total number of observed genotypes.

For the genotype correlation between the observed and imputed minor allele doses, the formula was as follows:

$$Imputed\;dosage=0*P\left(AA\right)+1*P\left(AB\right)+2*P\left(BB\right)$$

$$R^{2}=Cor^{2}\left(Imputed\;dosage,Observed\;dosage\right)$$

### 5.3 Estimation of Genetic Parameters

To understand the fundamental characteristics of semen quality traits, we estimated the genetic parameters (including heritability, repeatability, and genetic correlations) for each semen trait within each population. We first estimated the heritability and repeatability using a single‐trait repeatability model by the DMUAI module of DMU (Version 6, Release 5.2) [59]. The model was as follows:

y=Xb+Za+Wpe+e

where ***y*** is the semen quality phenotype. ***b*** is the vector of fixed effects, including the year of semen collection and season of semen collection. ***a*** ∼*N* (0, $\;A\sigma_{a}^{2}$) is the additive genetic effect, $\sigma_{a}^{2}$ is pedigree‐based additive genetic variance, and ***A*** is the pedigree‐based relationship matrix. ***pe*** ∼*N* (0, $\;I\sigma_{pe}^{2})$ is the vector of the permanent environment effect, $\sigma_{pe}^{2}$ is the permanent environment variance. ***e*** ∼*N* (0, $\;I\sigma_{e}^{2})$ is the vector of residual effect, $\sigma_{e}^{2}$ is the residual variance, and ***I*** is the identity matrix. ***X***, ***Z***, and ***W*** are the corresponding design matrices for the fixed effect ***b***, the additive genetic effect ***a***, and the permanent environment effect ***pe***.

Furthermore, we estimated the genetic correlation for all semen quality trait pairs using a double‐trait repeatability model. The model was as follows:

$$\left[\begin{array}{c} y_{1}\\ y_{2} \end{array}\right]=\left[\begin{array}{cc} \begin{array}{c} X_{1}\\ 0 \end{array} & \begin{array}{c} 0\\ X_{2} \end{array} \end{array}\right]\left[\begin{array}{c} b_{1}\\ b_{2} \end{array}\right]+\left[\begin{array}{cc} \begin{array}{c} Z_{1}\\ 0 \end{array} & \begin{array}{c} 0\\ Z_{2} \end{array} \end{array}\right]\left[\begin{array}{c} a_{1}\\ a_{2} \end{array}\right]+\left[\begin{array}{cc} \begin{array}{c} W_{1}\\ 0 \end{array} & \begin{array}{c} 0\\ W_{2} \end{array} \end{array}\right]\left[\begin{array}{c} pe_{1}\\ pe_{2} \end{array}\right]+\left[\begin{array}{c} e_{1}\\ e_{2} \end{array}\right]$$

where $\left[\begin{array}{c} y_{1}\\ y_{2} \end{array}\right]$ is the vector of phenotypic value for semen quality traits. $\left[\begin{array}{c} b_{1}\\ b_{2} \end{array}\right]$ is the vector of fixed effects, including the year of semen collection and season of semen collection. $\left[\begin{array}{c} a_{1}\\ a_{2} \end{array}\right]∼N\left(0,\;A[\begin{array}{cc} \begin{array}{c} \sigma_{a1}^{2}\\ \sigma_{a21} \end{array} & \begin{array}{c} \sigma_{a12}\\ \sigma_{a2}^{2} \end{array} \end{array}]\right)$ is the vector of the additive genetic effect, $\left[\begin{array}{cc} \begin{array}{c} \sigma_{a1}^{2}\\ \sigma_{a21} \end{array} & \begin{array}{c} \sigma_{a12}\\ \sigma_{a2}^{2} \end{array} \end{array}\right]$ is the additive genetic variance‐covariance matrix, and ***A*** is the pedigree‐based relationship matrix. $\left[\begin{array}{c} pe_{1}\\ pe_{2} \end{array}\right]∼N\left(0,\;I[\begin{array}{cc} \begin{array}{c} \sigma_{pe1}^{2}\\ \sigma_{pe21} \end{array} & \begin{array}{c} \sigma_{pe12}\\ \sigma_{pe2}^{2} \end{array} \end{array}]\right)$ is the vector of the permanent environment effect, $\left[\begin{array}{cc} \begin{array}{c} \sigma_{pe1}^{2}\\ \sigma_{pe21} \end{array} & \begin{array}{c} \sigma_{pe12}\\ \sigma_{pe2}^{2} \end{array} \end{array}\right]$ is the permanent environment variance‐covariance matrix. $\left[\begin{array}{c} e_{1}\\ e_{2} \end{array}\right]∼N\left(0,\;I[\begin{array}{cc} \begin{array}{c} \sigma_{e1}^{2}\\ \sigma_{e21} \end{array} & \begin{array}{c} \sigma_{e12}\\ \sigma_{e2}^{2} \end{array} \end{array}]\right)$ is the vector of residual effect, $\left[\begin{array}{cc} \begin{array}{c} \sigma_{e1}^{2}\\ \sigma_{e21} \end{array} & \begin{array}{c} \sigma_{e12}\\ \sigma_{e2}^{2} \end{array} \end{array}\right]$ is the residual variance‐covariance matrix. $\left[\begin{array}{cc} \begin{array}{c} X_{1}\\ 0 \end{array} & \begin{array}{c} 0\\ X_{2} \end{array} \end{array}\right]$, $\left[\begin{array}{cc} \begin{array}{c} Z_{1}\\ 0 \end{array} & \begin{array}{c} 0\\ Z_{2} \end{array} \end{array}\right]$ and $\left[\begin{array}{cc} \begin{array}{c} W_{1}\\ 0 \end{array} & \begin{array}{c} 0\\ W_{2} \end{array} \end{array}\right]$ are the corresponding design matrices for the fixed effect $\left[\begin{array}{c} b_{1}\\ b_{2} \end{array}\right]$, additive genetic effect $\left[\begin{array}{c} a_{1}\\ a_{2} \end{array}\right]$ and permanent environmental effect $\left[\begin{array}{c} pe_{1}\\ pe_{2} \end{array}\right]$.

To explore the genetic correlation between semen quality traits and other complex traits, we utilized a two‐trait linear mixed model by the DMUAI module of DMU (Version 6, Release 5.2). The model was as follows:

$$\left[\begin{array}{c} y_{1}\\ y_{2} \end{array}\right]=\left[\begin{array}{cc} \begin{array}{c} X_{1}\\ 0 \end{array} & \begin{array}{c} 0\\ X_{2} \end{array} \end{array}\right]\left[\begin{array}{c} b_{1}\\ b_{2} \end{array}\right]+\left[\begin{array}{cc} \begin{array}{c} Z_{1}\\ 0 \end{array} & \begin{array}{c} 0\\ Z_{2} \end{array} \end{array}\right]\left[\begin{array}{c} a_{1}\\ a_{2} \end{array}\right]+\left[\begin{array}{c} e_{1}\\ e_{2} \end{array}\right]$$

The meaning of the formula was the same as the double‐trait repeatability model. For the semen quality traits, we utilized the estimated breeding values (EBVs) derived from a single‐trait repeatability model as phenotypes. The fixed effects of other traits included the year of birth and season of birth.

### 5.4 GWAS in Single Population

To detect genomic variants associated with the semen traits, we conducted GWAS in each single population using GMAT software (v1.01) [27], which could well utilize longitudinal data to conduct GWAS using efficient multivariate analysis algorithms. We performed GWAS by using the repeatability model for each semen trait within each population as follows:

y=Xb+xc+wβ+Zg+Wpe+e

where ***y*** is a vector of phenotypes for semen quality traits. ***b*** is a vector of fixed effects including the year of semen collection and season of semen collection. ***c*** indicate the covariates, including the first five genotype principal components. ***x*** indicates the regression coefficient of covariates. ***β*** is the effect of SNP and ***w*** is a vector of SNP genotypes assigned a value of 0, 1, or 2 for AA, Aa, and aa, respectively. ***g*** ∼*N* (0, $\;G\sigma_{g}^{2}$) is a vector of additive genetic effect, ***G*** is the genomic relationship matrix built with the method of VanRaden [60], and $\sigma_{g}^{2}$ is the additive genetic variance. ***pe*** ∼*N* (0, $\;I\sigma_{pe}^{2})$ is the vector of permanent environmental effect, $\sigma_{pe}^{2}$ is the permanent environment variance. ***e*** ∼*N* (0, $\;I\sigma_{e}^{2})$ is the vector of residual effect, $\sigma_{e}^{2}$ is the residual variance, and ***I*** is the identity matrix. ***X***, ***Z*** and ***W*** are the corresponding design matrices for the fixed effect ***b***, additive genetic effect ***g*** and permanent environment effect ***pe***.

### 5.5 Meta‐GWAS

To ensure the quality of each GWAS summary statistics derived above, we implemented quality control using EasyQC (v23.8) [32]. Briefly, we standardized the individual GWAS summary statistics and utilized the filelevel_qc.ecf and metalevel_qc.ecf with default parameter settings to conduct the quality control in the file‐level and meta‐level, separately. After quality control, we performed the meta‐analysis for each semen quality trait across four breeds using METAL (version released on 2011‐03‐25) [33]. We utilized an inverse‐variance weighted fixed‐effects model implemented with parameters “SCHEME STDERR” and “GENOMICCONTROL ON”. To ensure the reliability of the discovery of GWAS meta‐analysis, we conducted quality control with (1) the variants that existed in lower than two populations, (2) the sample size of the corresponding variant lower than 30% and (3) the range (maximum‐minimum) of reference allelic frequency > 0.6 across populations for the same trait [61].

#### 5.5.1 Conditional Analysis and the Definition of QTL

To obtain the independent signals within each QTL, we performed the conditional analysis in all the individual GWAS and meta‐GWAS results using GCTA‐COJO [28]. We conducted conditional analysis for each individual GWAS with the corresponding genotypes as the linkage disequilibrium (LD) reference panel. Furthermore, we combined genotypes from all populations as the LD reference panel (N = 11 528) to conduct conditional analysis for meta‐GWAS. We considered the optional parameters: –*maf 0.01, –cojo‐slct, –cojo‐p 1e‐5* in conditional analysis. To define QTL, we first calculated the LD (r^2^) between the independent variant and the variants located in the region extending 1 Mb up‐ and down‐stream. After that, we defined the QTL region based on the high LD (*r* ^2^ ≥ 0.6) with independent variants. The LD reference panel used to define QTL was the same as the conditional analysis did. We then considered genes overlapping GWAS loci with at least one base pair as the candidate genes using bedtools [62].

#### 5.5.2 Gene‐based Association Analysis

To identify the potential genes associated with semen quality traits, we incorporated the GWAS summary statistics with genotype data derived from multi‐breed populations to conduct the summary‐based association analysis using MAGMA [37]. We set the Bonferroni correction (including suggestive significant threshold *p* = 1 / 22885 (total number of genes) = 4.37 × 10^−5^ and genome‐wide significant threshold *p* = 0.05 / 22885 (total number of genes) = 2.18 × 10^−6^) as a significant threshold.

#### 5.5.3 Functional Annotation for the Significant Variants and Candidate Genes

To identify the functional impacts of the semen quality‐associated variants, we annotated the genome location of suggestive (*p* < 1.00 × 10^−5^) significant variants using SNPEff [63] with *sus scrafa* 11.1 (version 110) and bedtools. To validate the QTL discovered in this study, we downloaded the Pig QTL list from PigQTLdb (release 52) [29], and extracted the 113 semen quality trait‐related QTL. By combining them based on the QTL regions located within 1 Mb, we obtained 40 independent QTL associated with the semen quality in PigQTLdb. Finally, we investigated the overlap between QTL discovered in this study and the QTL derived from PigQTLdb using bedtools.

To explore the biological and cellular function of the candidate genes discovered, we implemented the functional annotation for the candidate genes using David [64, 65]. We combined the list of candidate genes derived from the individual GWAS and meta‐analysis and conducted the functional annotation based on GO terms, KEGG pathways, and INTERPRO. Finally, we set the FDR < 0.05 as the significant threshold to obtain significantly enriched pathways.

#### 5.5.4 QTL Heritability Estimation

To estimate the QTL heritability, we first obtained the EBVs of semen quality traits because of the multiple records for each individual. We then utilized the two‐component linear mixed model to estimate the QTL heritability for each trait.

To obtain the adjusted phenotypes of semen quality traits, we first estimated the EBVs based on the pedigree information using a repeatability model using DMUAI and DMU4 module by DMU software [v5.2] [59]. The repeatability model was as follows:

y=Xb+Za+Wpe+e

where ***y*** was the vector of semen quality trait. ***b*** was the fixed effect, including the collective year and collective season. $a∼N(0,\;A\sigma_{a}^{2})$ was the vector of the additive genetic effect. $\sigma_{a}^{2}$ and ***A*** represented the additive genetic variance and pedigree‐based relationship matrix, respectively. $pe∼N(0,\;I\sigma_{pe}^{2})$ was the vector of permanent environmental effect. $\sigma_{pe}^{2}$ And ***I*** was the permanent environment variance and identity matrix. $e∼N(0,\;I\sigma_{e}^{2})$ was the vector of residual effect. $\sigma_{e}^{2}$ was the residual variance. the ***X***, ***Z*** and ***W*** was the design matrix for the ***b***, ***a***, and ***pe***.

After that, we calculated the adjusted phenotypes using the EBVs and residuals. The format was as follows:

$$y^{*}=EBV+\bar{e}$$

where ***y*** * were the adjusted phenotypes, $\bar{e}$ represented the average of residuals for each individual.

To explore the heritability explained by the QTL derived from GWAS meta‐analysis, we estimated the additive genetic variance of QTL using a two‐component linear mixed model. For the QTL heritability, we utilized the SNP located in the QTL regions to estimate the genetic variance. The description of the model was as follows:

$$y^{*}=Zg+Zg_{F}+e$$

where the ***y*** ^*^ were the adjusted phenotypes. $g∼N(0,\;G\sigma_{g}^{2}$) was a vector of additive genetic effect, ***G*** was the genomic relationship matrix based on WGS data, and $\sigma_{g}^{2}$ was the additive genetic variance. $g_{F}∼N(0,\;G_{F}\sigma_{g_{F}}^{2}$) was the vector of the genetic effect of the genomic feature. $\sigma_{g_{F}}^{2}$ And ***G***_***F***_ were the genetic variance of the genomic feature and the genomic relationship matrix established by the genomic feature. $e∼N(0,\;I\sigma_{e}^{2})$ was the vector of residual effect. $\sigma_{e}^{2}$ was the residual variance. To further explore the importance of regulatory elements/variants, we estimated the per SNP heritability explained by the regulatory elements/variants using a two‐component linear mixed model.

For the regulatory elements, we downloaded chromatin states across 14 tissues from the FAANG project [41]. We utilized the SNP located in the corresponding regulatory element as the second component to estimate the genetic variance and calculate the per SNP heritability. For the regulatory variants, we first obtained the independent variants for the breeding population with the parameter *–indep‐pairwise 500 50 0.25* using PLINK v1.90 [66]. Then, we downloaded the independent e/sQTL for each tissue from PigGTEx. Next, we combined the independent regulatory variants as the second component to estimate the genetic variance and calculate the per SNP heritability. We utilized the format: per SNP heritability = (heritability explained by the genomic feature) / (number of variants located in the corresponding genomic element) to calculate the per SNP heritability.

### 5.6 Integrative Genomics Analysis

To understand the molecular regulation of gene expression on semen quality traits in pigs, we utilized the PigGTEx data to conduct TWAS and colocalization analyses to detect the potential regulatory genes across 28 pig tissues.

#### 5.6.1 Transcriptome‐wide Association Study (TWAS)

To detect the significant association between gene expression and semen quality traits, we downloaded the TWAS models from the PigGTEx resource to implement TWAS with the GWAS meta‐analysis summary statistics using S‐PrediXcan [67]. We considered genes with *p*‐values below the Bonferroni correction threshold (*p* = 0.05/ the number of genes for each tissue) as significant genes.

#### 5.6.2 Colocalization Analysis

To detect the potential regulatory genes of semen traits, we utilized summary statistics from both eQTL mapping summary statistics and meta‐GWAS summary statistics to conduct colocalization analysis using the *coloc* R package (v5.2.2) [67]. Based on the independent variants derived from conditional analysis, we extracted the independent variants with extending up‐ and down‐stream of 1 Mb from the meta‐GWAS summary statistics as the GWAS inputs. Correspondingly, we extracted the summary statistics of eQTL mapping from the same GWAS windows as the eQTL inputs. Next, we conducted the colocalization analysis for each gene with the default parameters by *coloc* R package. Finally, we set the posterior probability 4 (PP4) of 0.75 as the significant threshold to define candidate genes.

### 5.7 Comparative Analysis Between Pigs, Cattle, and Humans

#### 5.7.1 Cattle Data and Human Data

For the GWAS dataset of cattle, we downloaded the GWAS summary statistics of 36 dairy traits from Jiang's study [68]. In addition, we collected GWAS summary statistics of five semen quality traits in cattle using the same approach as described above. The detail of this cattle population was described in the ref. [8]. For the TWAS dataset of cattle, we downloaded the TWAS result of cattle reproductive traits from CattleGTEx [22]. For the semen traits of cattle, we downloaded the 24 TWAS models from cattleGTEx to conduct the TWAS for each tissue. We considered genes with *P‐*values below the Bonferroni correction threshold (*p* = 0.05/ the number of genes for each tissue) as significant genes. For the GWAS dataset of humans, we downloaded GWAS summary statistics of 25 traits/diseases from fastGWA [69, 70].

#### 5.7.2 Comparison Analysis of TWAS Between Cattle and Pigs

To explore the connection between semen quality traits and other types of traits between cattle and pigs, we first extracted the one‐to‐one orthologous genes between pigs and cattle. Then, we downloaded the TWAS of cattle complex traits from the CattleGTEx resource [22]. Next, we extracted the one‐to‐one orthologous genes derived from Ensembl v110 in the TWAS of pigs and cattle. Then, we calculated Pearson's correlation of the absolute z‐score of orthologous genes between semen quality traits of pigs and all the cattle traits across 14 tissues. Finally, we considered genes with *P*‐values below the Bonferroni correction threshold (*p* = 0.05/ the number of genes for each tissue) as significant genes.

#### 5.7.3 Heritability Enrichment Analysis in Human Complex Traits/Diseases

To explore the potential function of these semen quality‐associated genes in human complex traits/diseases, we first extracted all the significant genes from TWAS and colocalization in pigs. Then, we extended 100 kb for the up‐ and down‐stream of the genes. Next, we utilized the LiftOver tools (https://genome.ucsc.edu/cgi‐bin/hgLiftOver) to map the regions from pig (sus scrafa 11.1) to the human genome (GRCh37/hg19). To implement the heritability enrichment analysis, we downloaded the European reference panel derived from 1000 human genome project, and calculated the LD scores with default parameters using LDSC [52]. We standardized the summary statistics using the *munge_sumstats.py* script and partitioned heritability for each of 25 complex traits/diseases to obtain the heritability enrichment on the above orthologous regions.

## Author Contributions

Z.Z.(SCAU), L.F., Y.Z., Z.Z.(ZJU), Y.Y., and C.N. performed in study design. Q.L., Z.Z., T.L., Z.X., C.W., X.Z., J.Z., H.C., Z.Z., X.L., Y.T., and S.C. performed in genotype and phenotype data processing. Q.L. and X.C. performed in genetic parameter estimation. Q.L. and X.C. performed in individual GWAS and meta‐analysis. Q.L., X.C., T.L., and X.C. performed in integration between GWAS and multi‐omics data. Q.L., Y.T., and S.C. performed in comparison between mammals. Q.L. performed in writing of the original draft. Z.Z.(SCAU), L.F., Z.Z.(ZJU), W.A., and Y.G. performed in writing of the review and editing. C.N. performed in methodology development. Z.Z.(SCAU), Y.Z., Z.Z.(ZJU), Y.Y., Y.P., and Q.W. performed in data contribution for GWAS. Z.Z.(SCAU) and J.L. performed in funding support.

## Funding

This work was supported by the fundings from National Key R&D Program of China (2022YFF1000900) and the Earmarked Fund for China Agriculture Research System (CARS‐35)

## Conflicts of Interest

The authors declare no conflicts of interest.

## Supporting information




**Supporting File 1**: advs73694‐sup‐0001‐SuppMat.docx.

**Supporting File 2**: advs73694‐sup‐0002‐Supplementary_tables.xlsx.

## Data Availability

The data that support the findings of this study are available on request from the corresponding author. The data are not publicly available due to privacy or ethical restrictions.
