# Supplementary material for: Meta‐GWAS of Pig Semen Quality Traits Reveals Conserved Genes Regulating Mammalian Fertility
Source: Adv Sci (Weinh). 2026 Jan 8;13(16):e15203. doi: 10.1002/advs.202515203 (PMC13042736; doi:10.1002/advs.202515203)
Supplement: Supplementary file 1 — Supporting File 1: advs73694‐sup‐0001‐SuppMat.docx. [file ADVS-13-e15203-s002.docx]

**Supporting information**


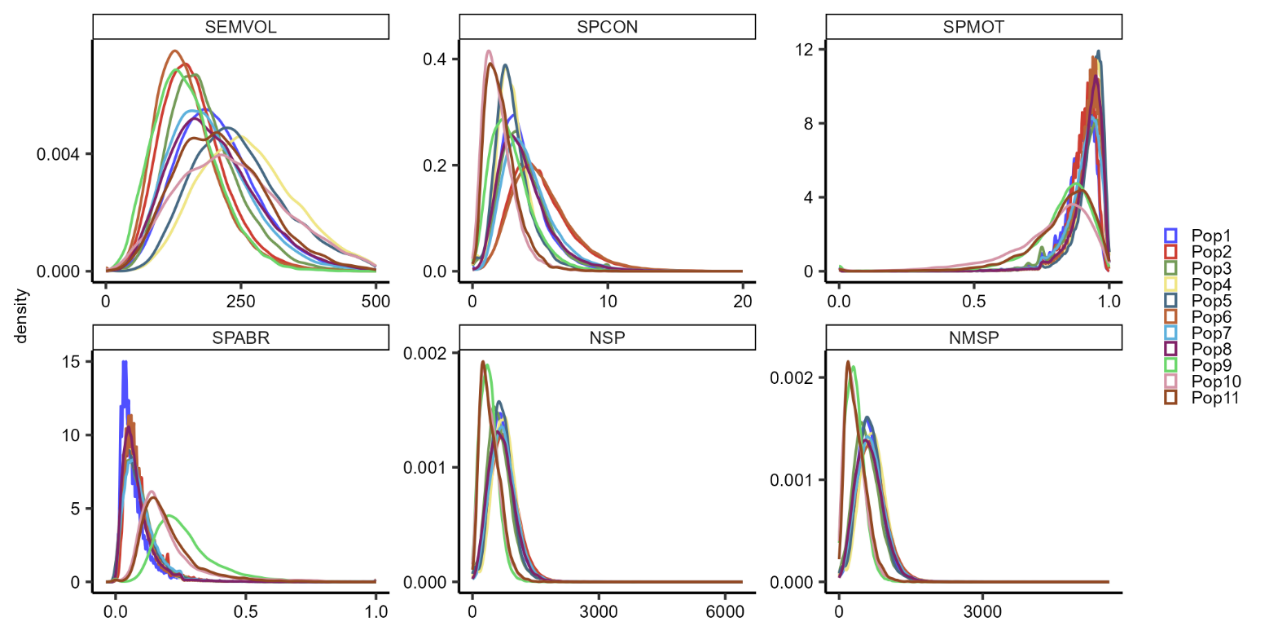


**Figure S1 The density distribution of six semen quality traits across 11 populations.**


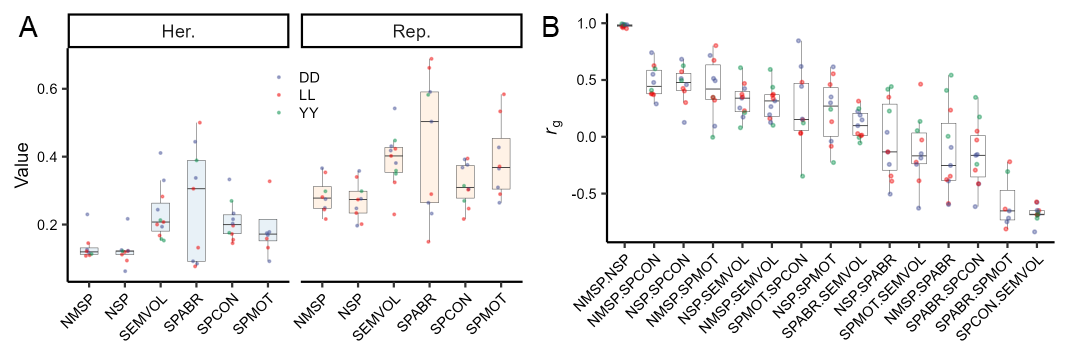


**Figure S2 The genetic parameters across populations for semen quality in pigs.**

(A) The distribution of narrow-sense heritability and repeatability for six semen quality traits. Each point indicates the narrow-sense heritability and repeatability for each population and each trait. Her., heritability. Rep., represents the repeatability. DD, Duroc. LL, Landrace. YY, Yorkshire.

(B) The genetic correlation between semen quality traits. The boxes are sorted by the mean of genetic correlations for each trait pairs. Each point indicated the genetic correlation for each population of each trait pair.


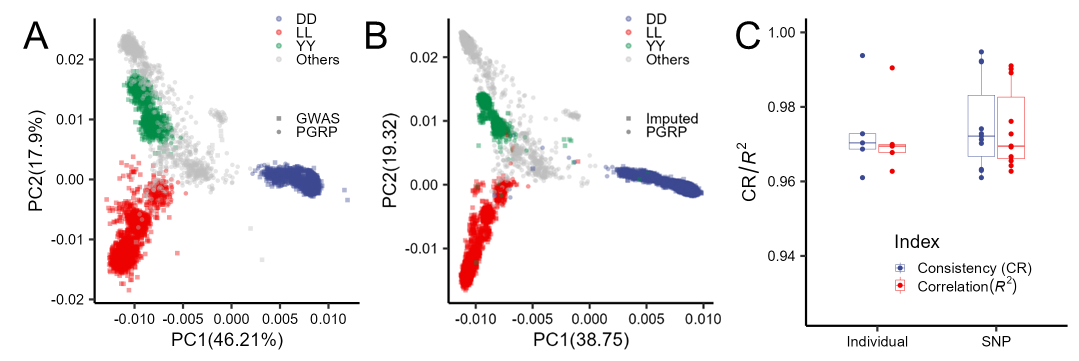


**Figure S3 The evaluation of the accuracy of genotype imputation.**

(A-B) The PCA for genotype imputation reference panel (PGRP) and all GWAS population before and after genotype imputation.

(C) The performance of genotype imputation, including consistency (CR) and correlation (*R^2^*), across all GWAS populations in the individual and SNP levels.


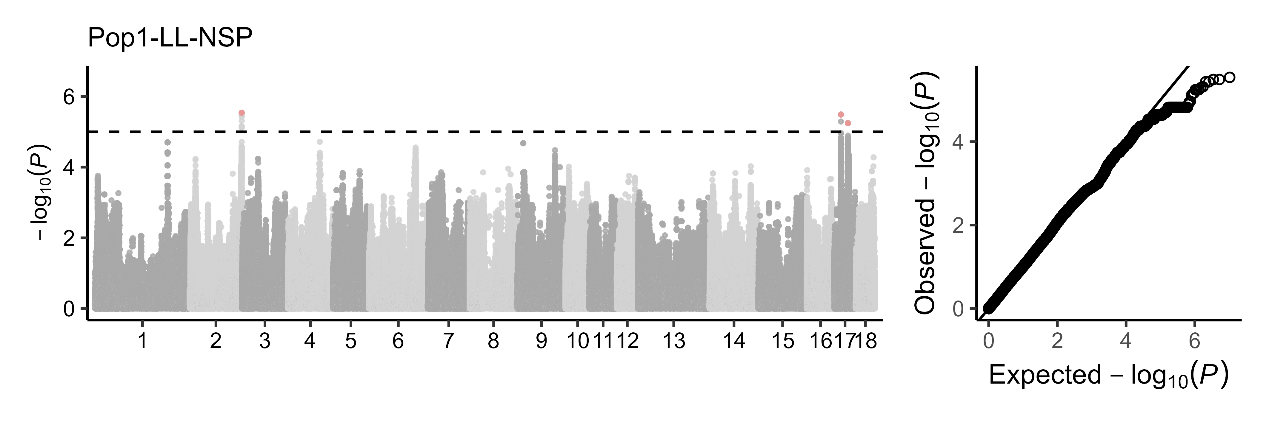


**Figure S4 The Manhattan plot and QQPlot for each trait in each population.**

The x-axis indicates the chromosomes. The dashed line indicates the suggestive significant threshold (*P* < 1.00×10^-5^). The light red points represent the independent variants deriving from the conditional analysis using GCTA-COJO.


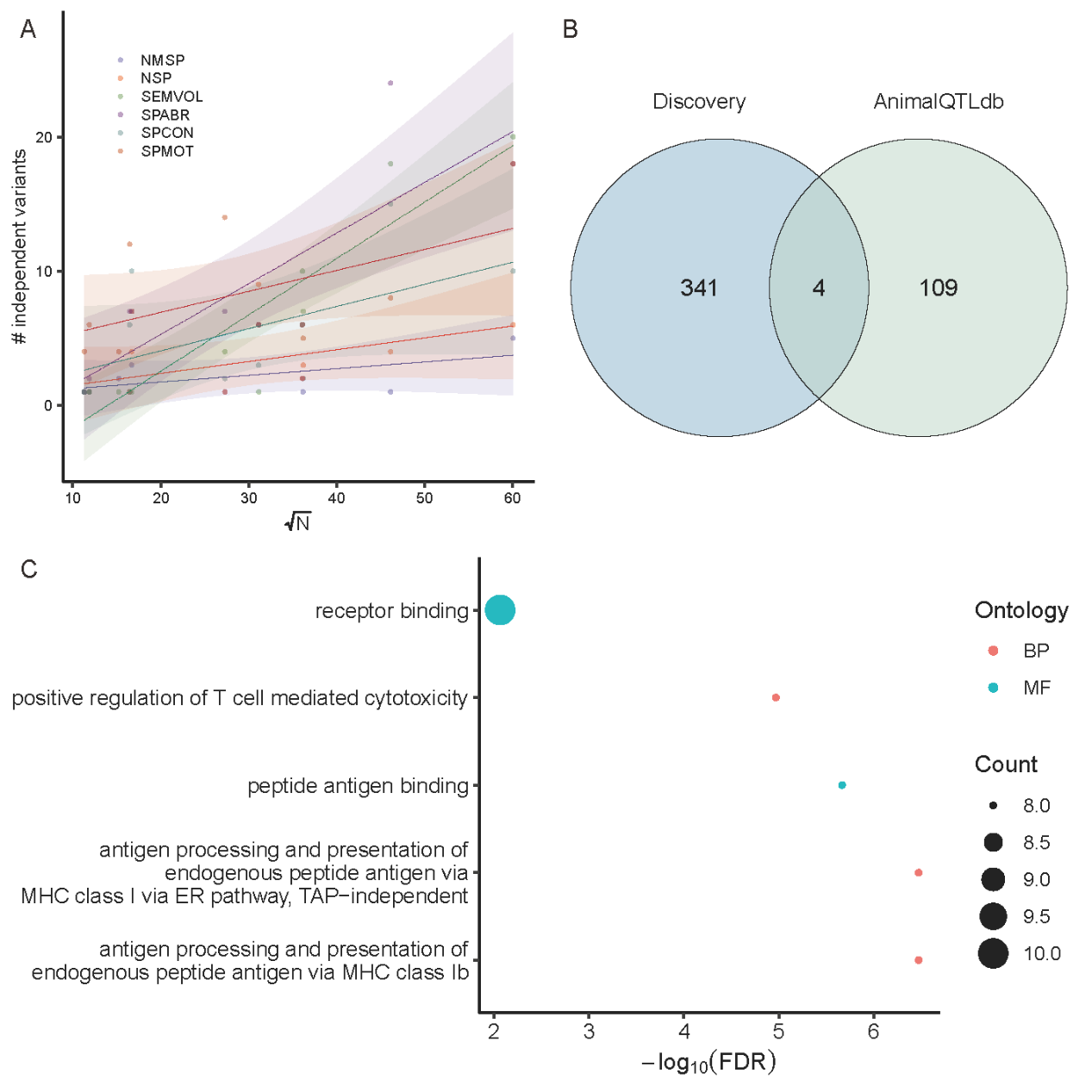


**Figure S5 The traditional post-GWAS analysis for the individual GWAS.**

(A) The correlation between sample size and number of independent variants across populations and traits. The lines are fitted using the *geom_smooth* function in *ggplot2* R packages. The background colors around the lines represent the 95% confidence intervals.

(B) The overlaps of QTL Derived from the individual GWASs (Discovery set, blue) and the Animal QTL database (Animal QTLdb, yellow).

(C) The functional enrichment (GO term) of 277 candidate genes passed genome-wide significant threshold discovered in individual GWAS. The colors represent the Ontology. The point sizes present the number of genes enriched in the GO terms. FDR, false discovery rate.


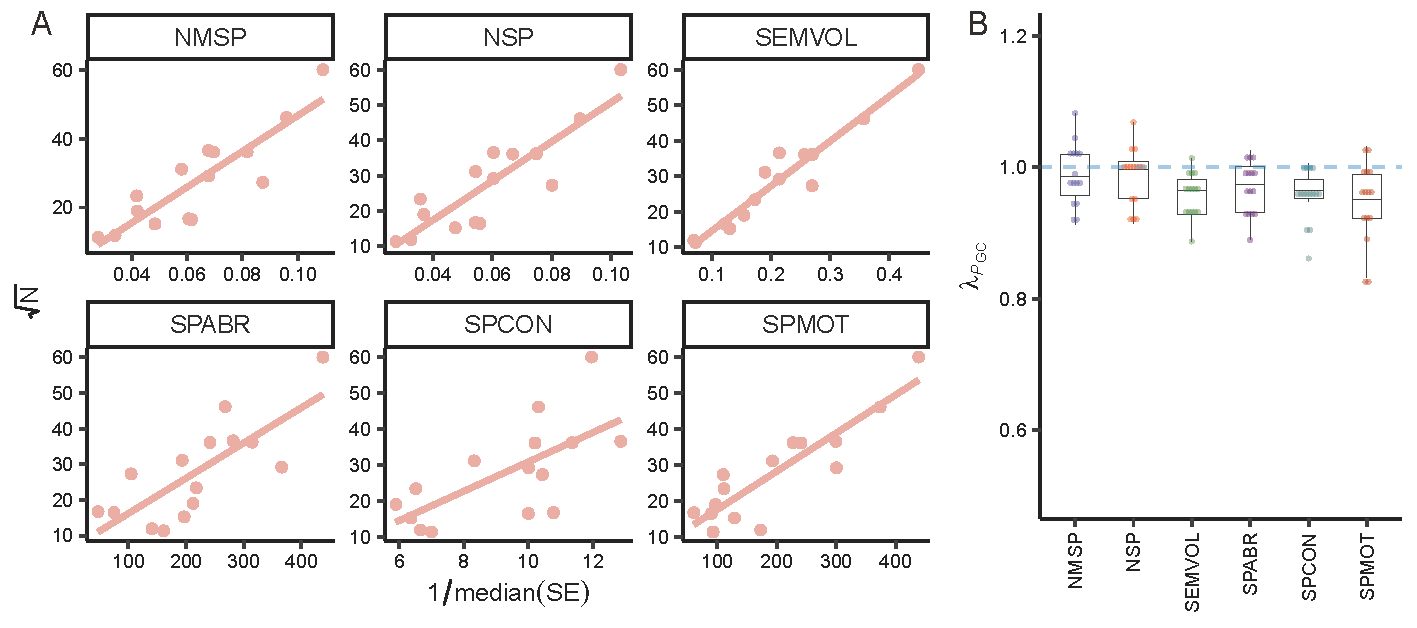


**Figure S6 The quality control of individual GWASs before GWAS meta-analysis.**

(A) The correlation between the sample size and the median of standard error. Each point represents each male trait in each population. The red line is fitted using the *geom_smooth* function in *ggplot2* R packages.

(B) The distribution of lambda (GC) for each semen quality trait. Each point represents each population. The blue dashed line indicates the lambda = 1.00.


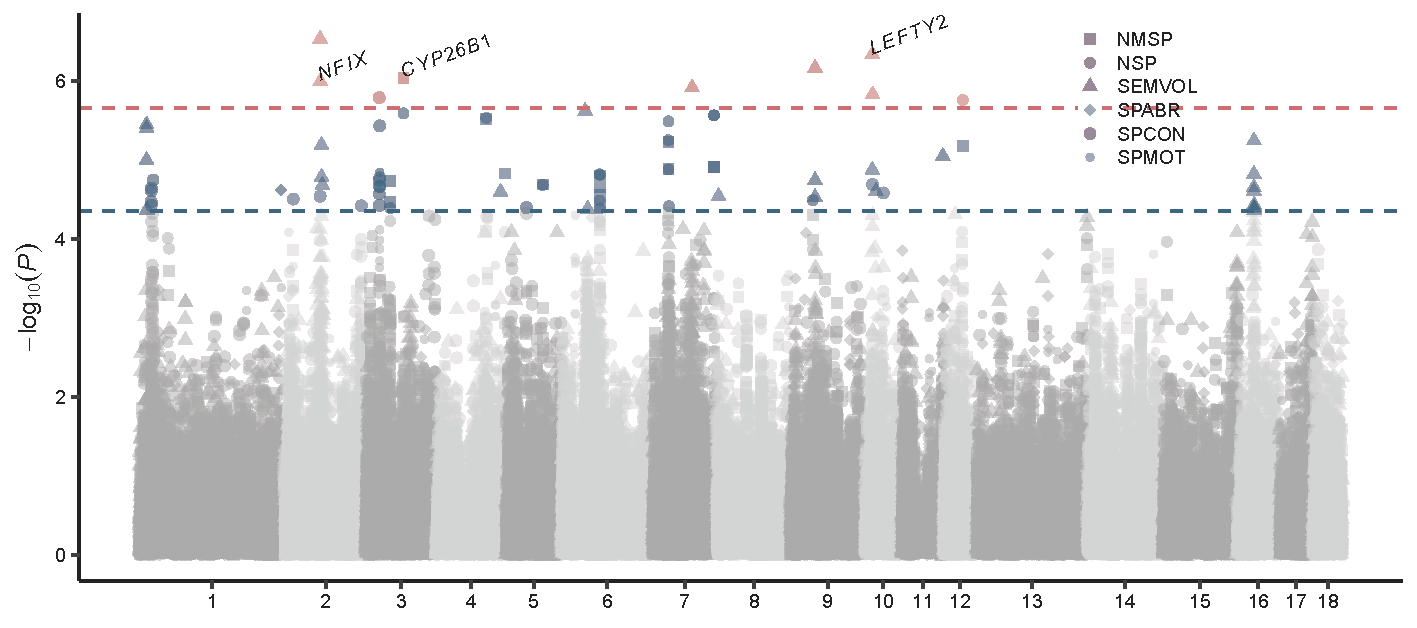


**Figure S7 Gene-based association analysis for six semen quality traits.**

The red and blue points represent the genome-wide and suggestive significant genes associated with semen quality traits. The shapes of points indicate the different traits. The red and blue dashed lines represent the genome-wide (*P* = 0.05/22885 = 2.18×10^-6^) and suggestive (*P* = 1/22885 = 4.37×10^-5^) significant threshold. The italic texts represent the gene names.


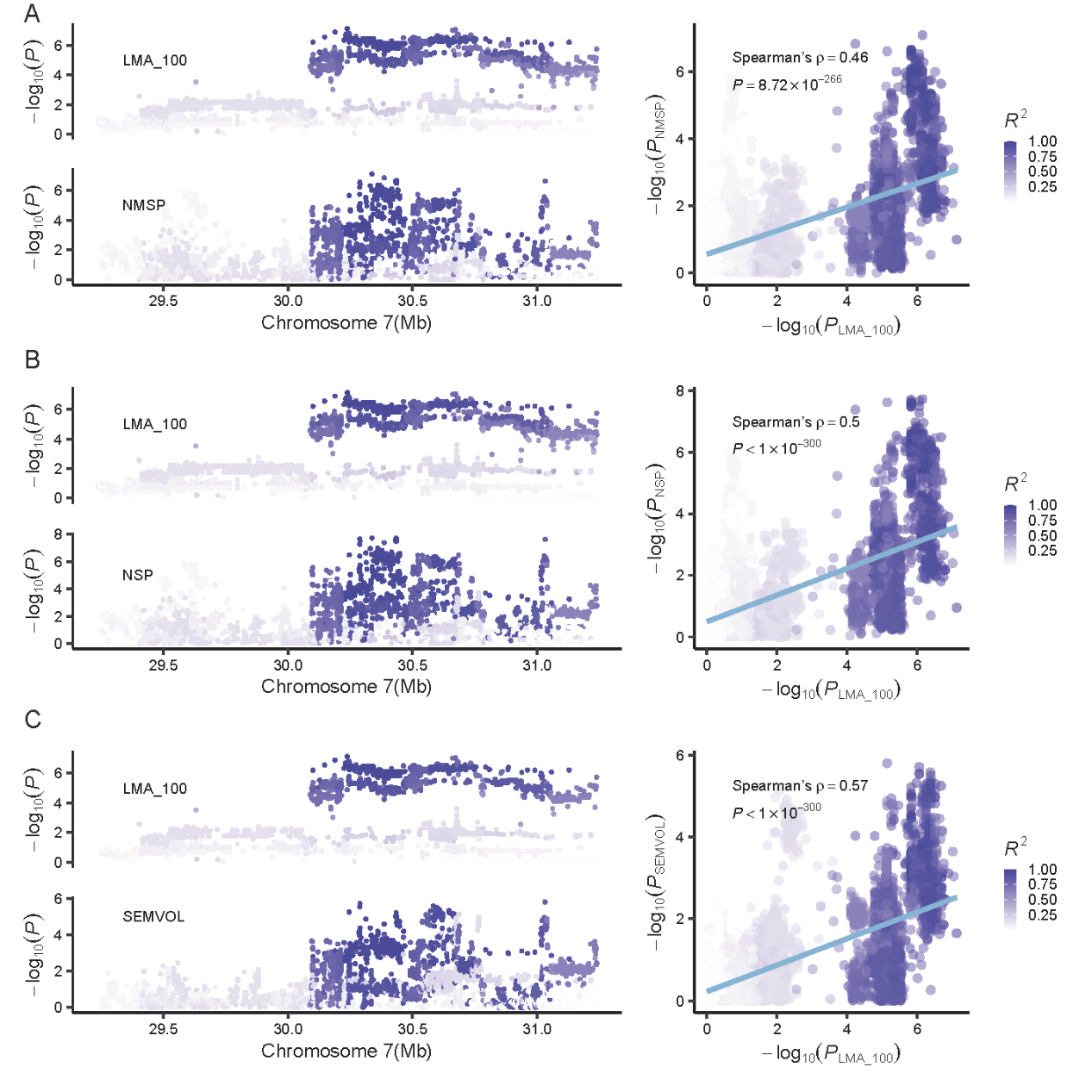


**Figure S8 The regional GWAS Manhattan plot on chromosome 7.** A-C corresponded to NMSP, NSP and SEMVOL with LMA_100, respectively.


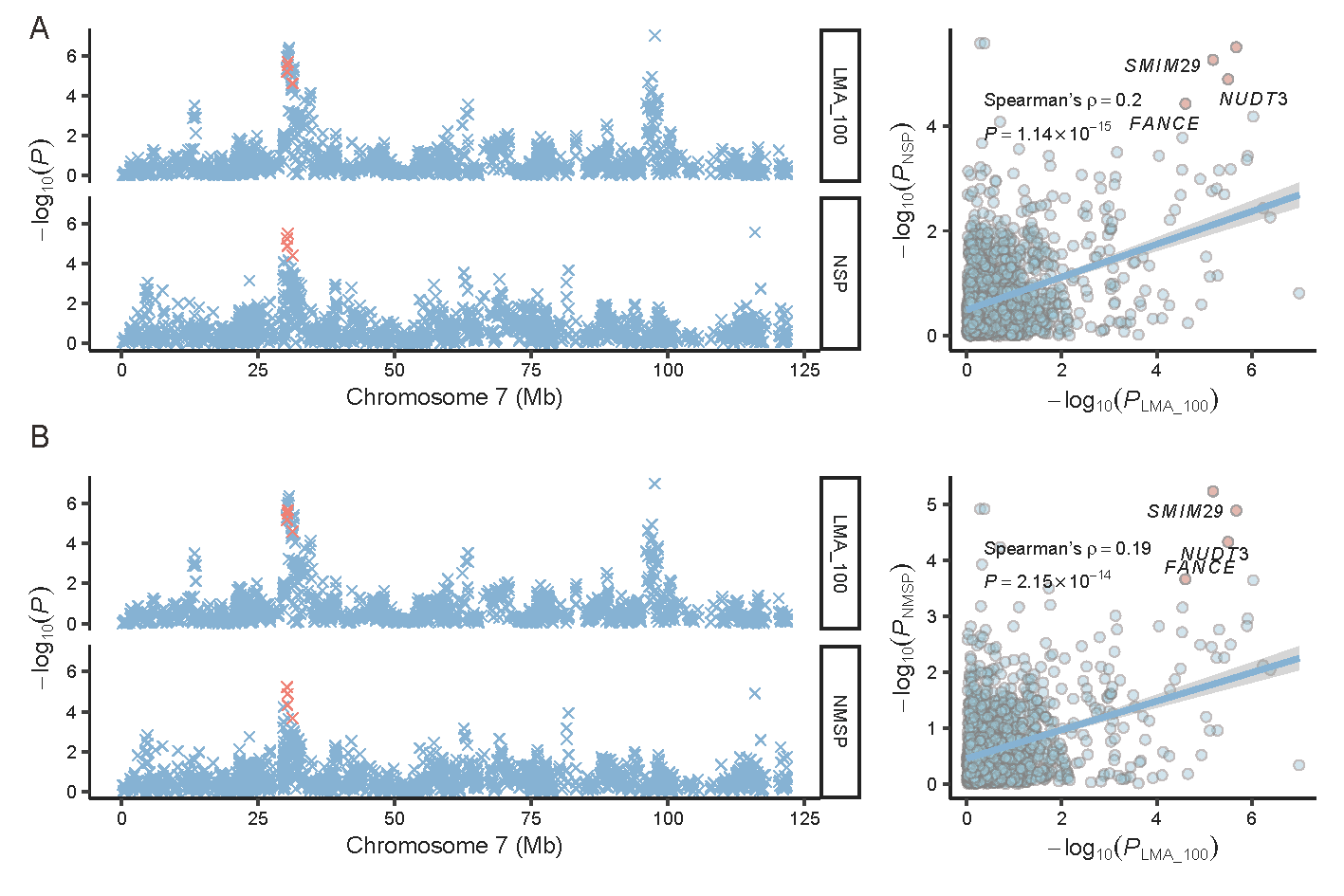


**Figure S9 The regional gene-based association analysis Manhattan plot on chromosome 7.** A-B corresponded to NSP and NMSP with LMA_100, respectively.


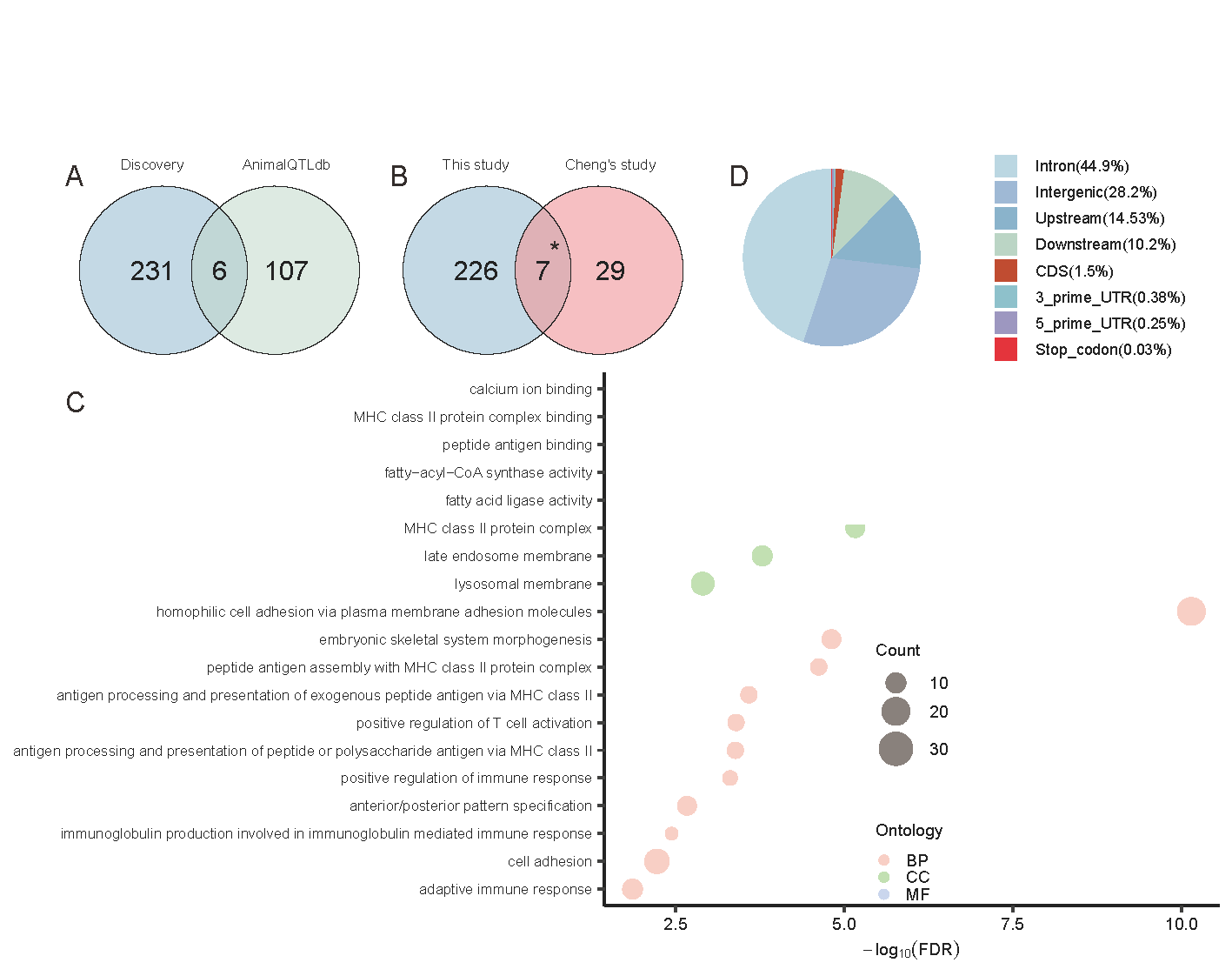


**Figure S10 The validation of QTL derived from the GWAS meta-analysis across six semen quality traits.** (A) The Venn plot demonstrated the overlap of QTL derived from GWAS meta-analysis (blue) and Pig QTLdb (green). (B) The overlapping of QTL between this study and Cheng’s study ^[13]^. The star indicated the significant enriched calculated by Fisher test. (C) The functional enrichment (GO term) of candidate genes discovered in GWAS meta-analysis. The colors represent the Ontology. CC, cell component. BP, biological process. MF, molecular function. The point sizes present the number of genes enriched in the GO terms. FDR, false discovery rate. (C) The genome annotation of suggestive significant variants across six semen quality traits. CDS, coding sequence. UTR, untranslated region.


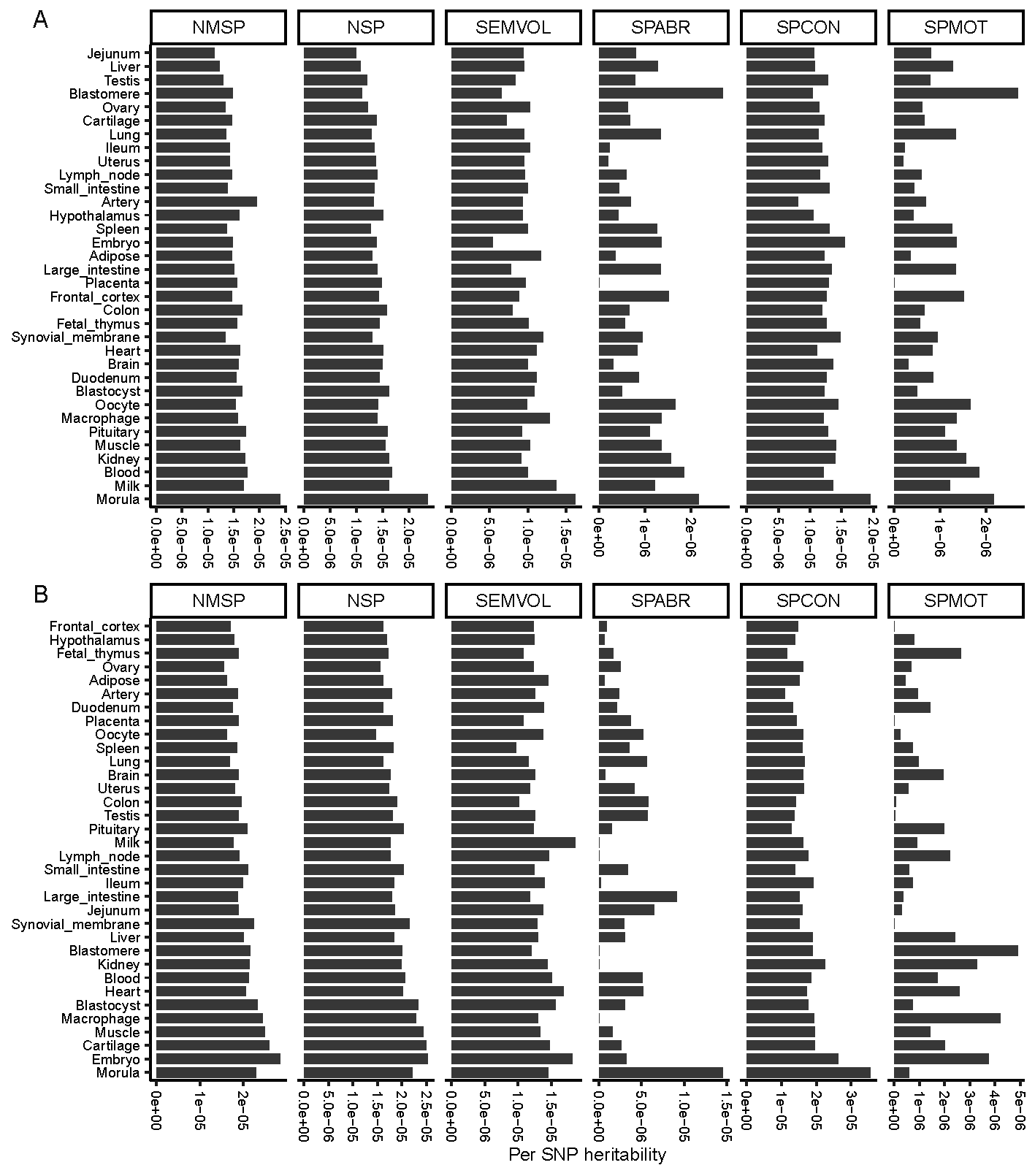


**Figure S11 The per SNP heritability explained by eQTL (A) and sQTL (B) within each tissue.**

The tissues were sorted by the mean of per SNP heritability across six semen quality traits.


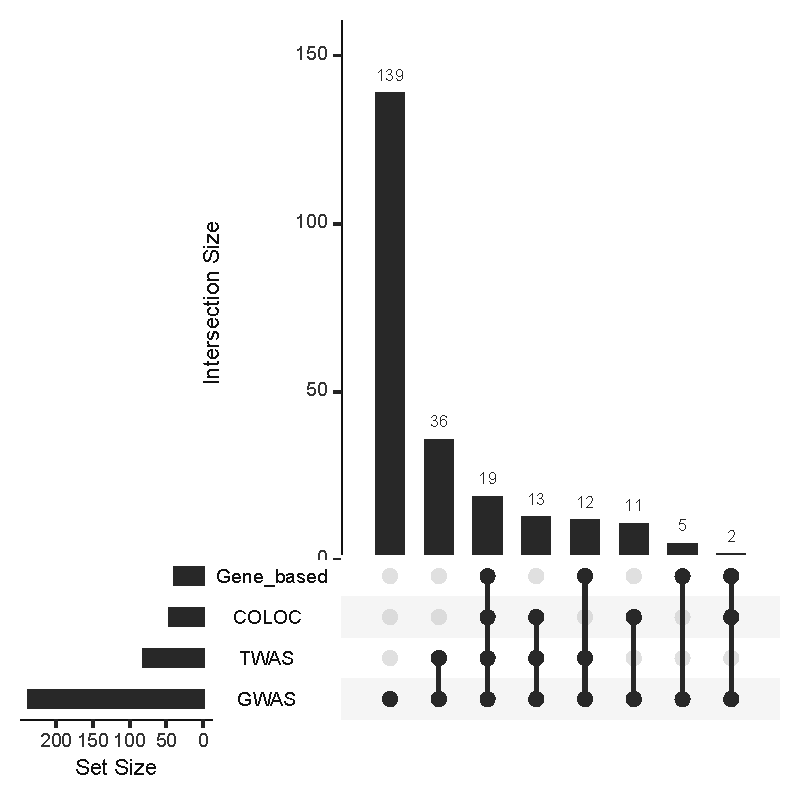


**Figure S12 The upsetR summary of post-GWAS in semen quality traits.**


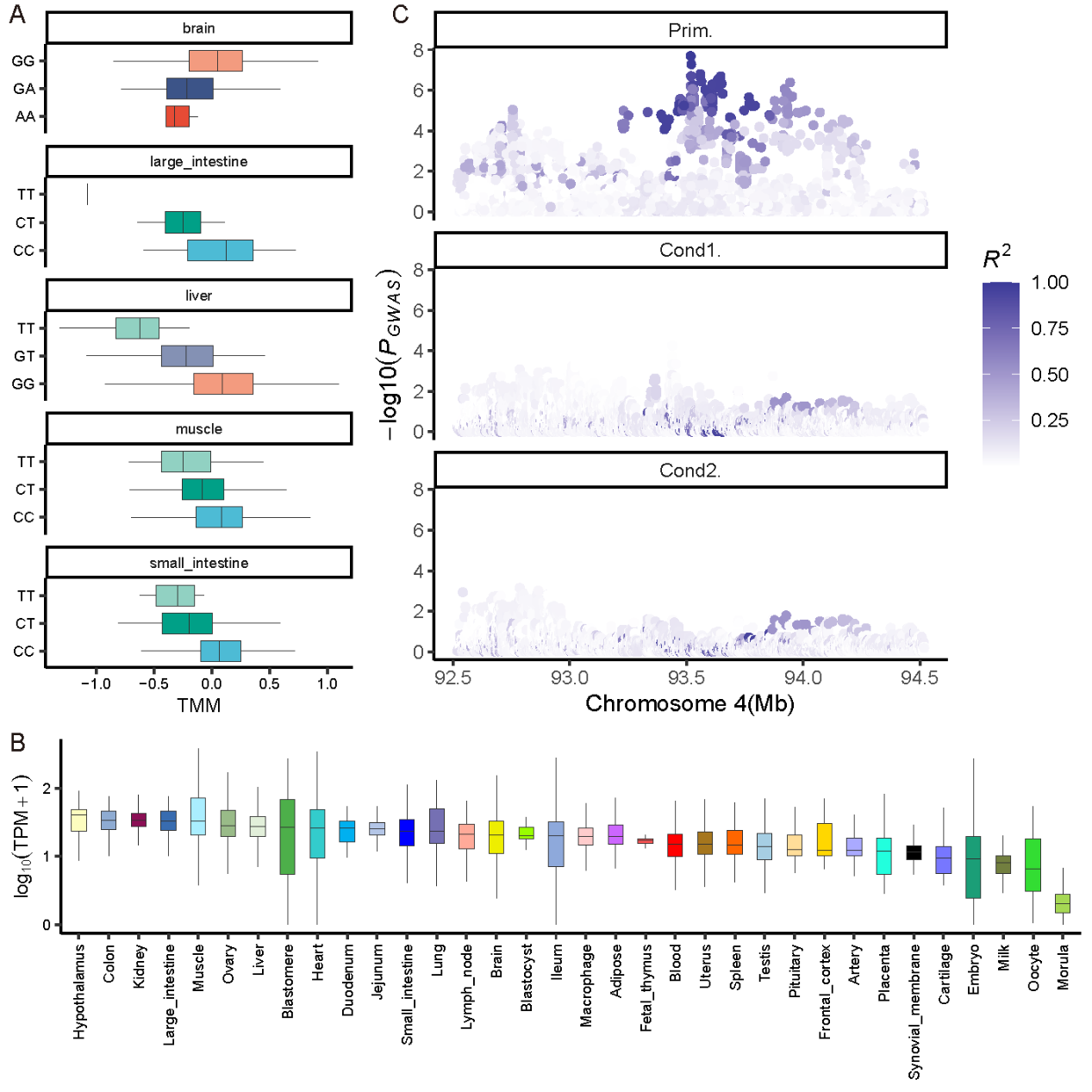


**Figure S13 The GWAS region with lead SNP on NAXE genes.**

(A) The gene expression of NAXE with different genotypes of top eQTL in five tissues (including the brain, large intestine, small intestine, muscle and liver).

(B) The Manhattan plot with conditional analysis of the GWAS loci of NSP with lead variant rs322657255. Prim. Indicates the primary GWAS loci. Cond1. represent the GWAS loci with conditional analysis for lead variant rs322657255. Cond2. represent the GWAS loci with conditional analysis for lead variant rs322657255 and secondary lead variant rs343458850.

(C) The expression of the *NAXE* gene across 34 tissues.


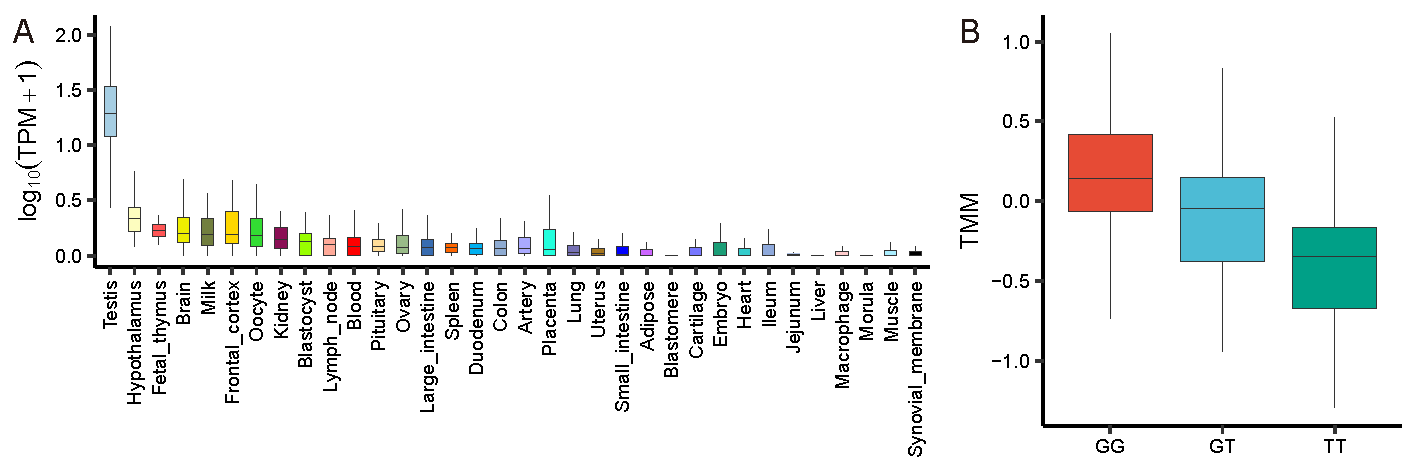


**Figure S14 The LEFTY2 genes.**

(A) The expression of *LEFTY2* gene across 34 tissues.

(B) The gene expression of the *LEFTY2* gene with different genotypes of top eQTL in testis.


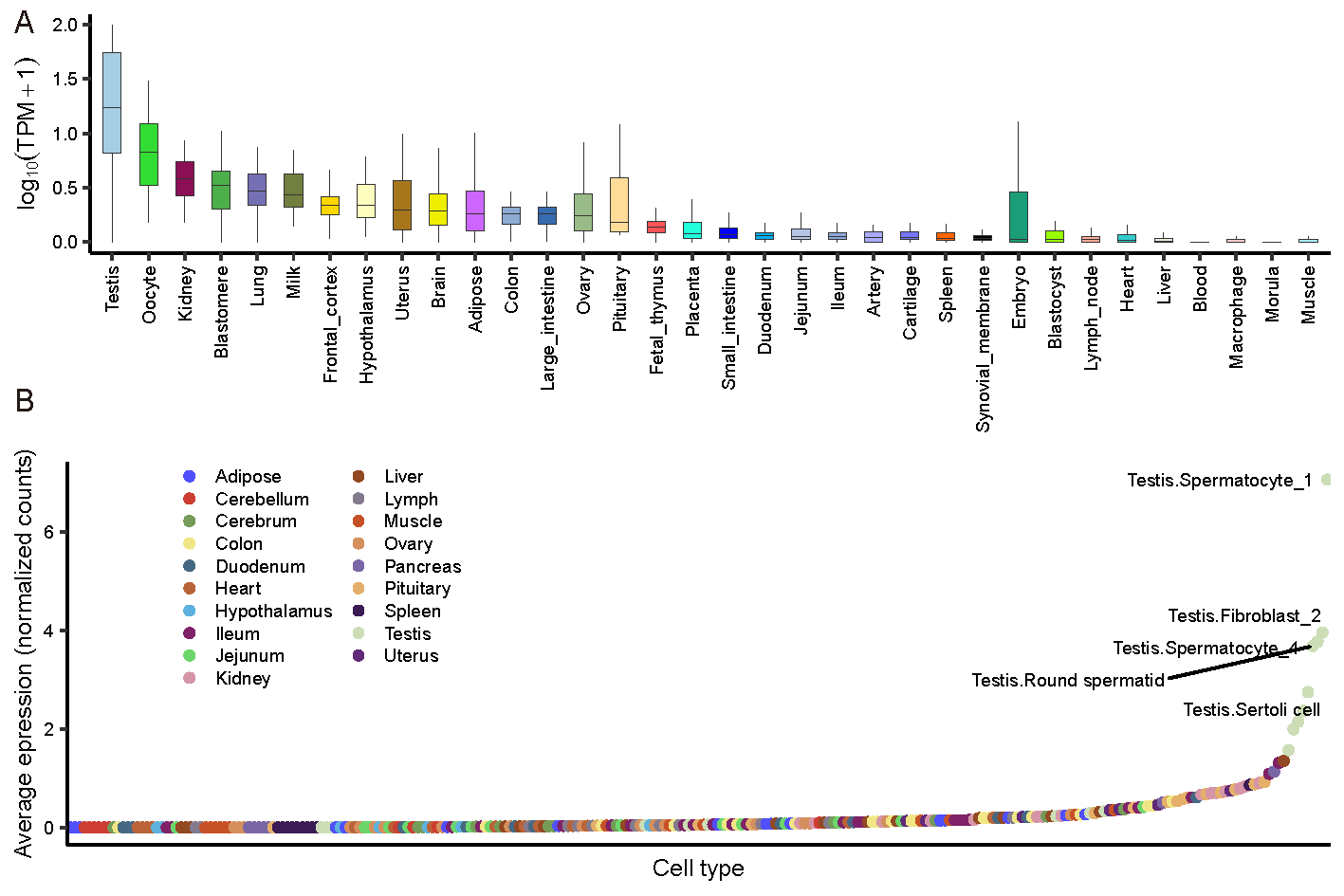


**Figure S15 *TTC29* gene.**

(A) The expression of the *TTC29* gene across 34 tissues.

(B) The expression of the *TTC29* gene across tissues and cell types.


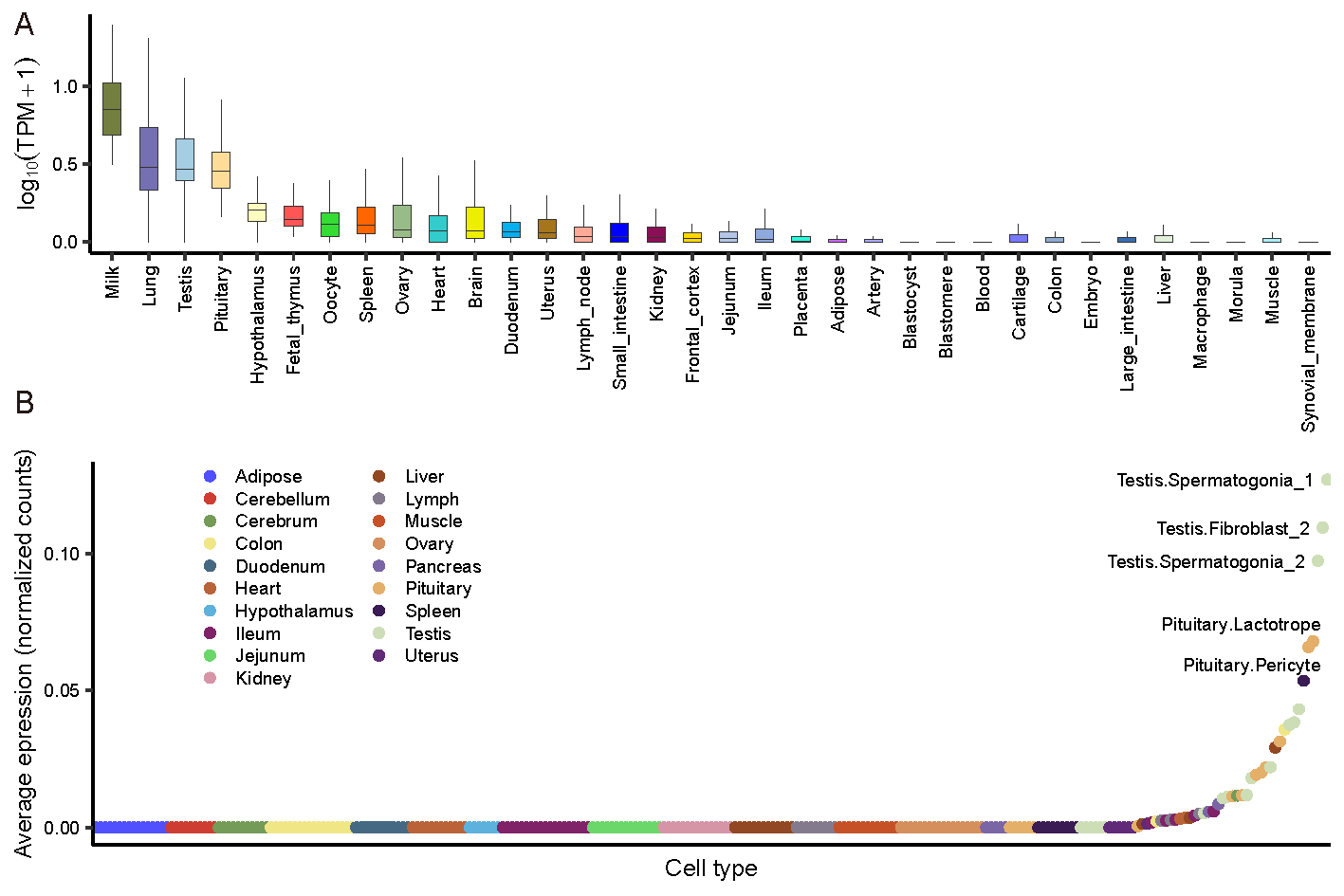


**Figure S16 *DMRTA1* gene.**

(A) The expression of the *DMRTA1* gene across 34 tissues.

(B) The expression of the *DMRTA1* gene across tissues and cell types.


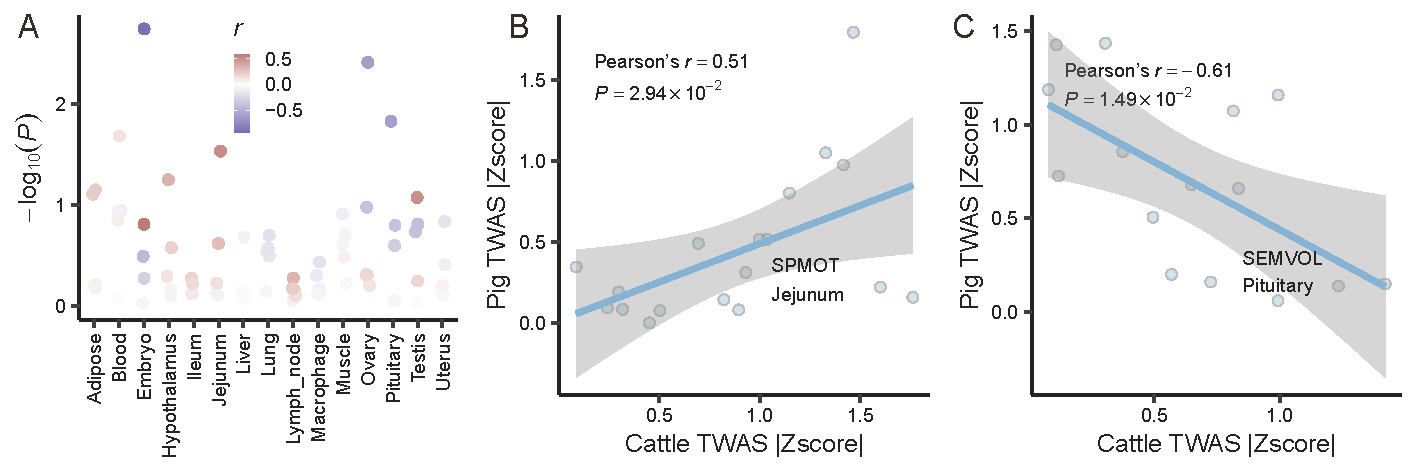


**Figure S17 The Pearson’s correlation of absolute z-score derived from TWAS for semen quality traits between cattle and pigs across 15 tissues.**

(A) The point color indicates the Pearson’s correlation.

(B-C) The example of trait-tissue pairs. (B) SPMOT with jejunum. (C) SEMVOL with pituitary.

Table S1 Phenotype summary of semen quality traits for each trait each population

Table S2 The genetic parameters for each population and semen quality trait

Table S3 The characteristics and summary of single-breed GWAS for each population and semen quality trait

Table S4 The list of QTL information with candidate genes derived from single-breed GWASs

Table S5 The functional annotation of candidate genes satisfied with genome wide significant threshold derived from single-breed GWASs

Table S6 The list of QTL information with candidate genes derived from GWAS meta-analysis

Table S7 The significant associated genes associated with semen quality traits originated from gene-based association analysis

Table S8 The functional annotation of candidate genes derived from GWAS meta-analysis

Table S9 The colocalized tissue-gene-traits pairs (posterior probability 4 > 0.75) with GWAS meta-analysis

Table S10 The significant tissue-gene-traits pairs derived from TWAS of GWAS meta-analysis

Table S11 The Pearson correlation of z-score derived from TWAS between cattle and pig
